# Intermittent Preventive Treatment (IPT): Its Role in Averting Disease-Induced Mortality in Children and in Promoting the Spread of Antimalarial Drug Resistance

**DOI:** 10.1007/s11538-018-0524-1

**Published:** 2018-10-31

**Authors:** Carrie A. Manore, Miranda I. Teboh-Ewungkem, Olivia Prosper, Angela Peace, Katharine Gurski, Zhilan Feng

**Affiliations:** 1 Los Alamos National Laboratory and New Mexico Consortium, Los Alamos, USA; 2 Department of Mathematics, Lehigh University, Bethlehem, USA; 3 Department of Mathematics, University of Kentucky, Lexington, USA; 4 Department of Mathematics and Statistics, Texas Tech University, Lubbock, USA; 5 Department of Mathematics, Howard University, Washington, USA; 6 Department of Mathematics, Purdue University, West Lafayette, USA

**Keywords:** Age structure, Malaria-induced deaths, *Plasmodium falciparum*, Intermittent preventative treatment, Holoendemic, Immunity

## Abstract

We develop an age-structured ODE model to investigate the role of intermittent preventive treatment (IPT) in averting malaria-induced mortality in children, and its related cost in promoting the spread of antimalarial drug resistance. IPT, a malaria control strategy in which a full curative dose of an antimalarial medication is administered to vulnerable asymptomatic individuals at specified intervals, has been shown to reduce malaria transmission and deaths in children and pregnant women. However, it can also promote drug resistance spread. Our mathematical model is used to explore IPT effects on drug resistance and deaths averted in holoendemic malaria regions. The model includes drug-sensitive and drug-resistant strains as well as human hosts and mosquitoes. The basic reproduction, and invasion reproduction numbers for both strains are derived. Numerical simulations show the individual and combined effects of IPT and treatment of symptomatic infections on the prevalence of both strains and the number of lives saved. Our results suggest that while IPT can indeed save lives, particularly in high transmission regions, certain combinations of drugs used for IPT and to treat symptomatic infection may result in more deaths when resistant parasite strains are circulating. Moreover, the half-lives of the treatment and IPT drugs used play an important role in the extent to which IPT may influence spread of the resistant strain. A sensitivity analysis indicates the model outcomes are most sensitive to the reduction factor of transmission for the resistant strain, rate of immunity loss, and the natural clearance rate of sensitive infections.

## Introduction

Malaria continues to be a burden in many parts of the world, especially in the African continent. An estimated 214 million new malaria cases (range 149–303 million) were reported worldwide in 2015, with Africa contributing the most, about 88%, followed by Southeast Asia and the Eastern Mediterranean region, each contributing 10% and 2%, respectively (World Health Organization 2015b). The estimated 2015 worldwide number of deaths was 438, 000, a decline from the 2012 estimates. Of these deaths, 90% came from the African region, 7% from Southeast Asia, and 2% from the Eastern Mediterranean region (World Health Organization 2014a, b, 2015b). Although malaria mortality rates are dropping (down by 60% worldwide between 2000 and 2015), many people still suffer the burdens of illness, infection, and death, with children under five more susceptible to these burdens. In fact, the 2015 globally estimated under five deaths was 306, 000 (World Health Organization 2015b). Thus, strategies for reducing infection and disease burden in infants and children, groups bearing the highest burden of the disease, are increasingly urgent. Intermittent preventive treatment (IPT) is one such strategy employed.

IPT is a preventative malaria control strategy used as a tool to reduce disease burden and death among infants, children, and pregnant women (Gosling et al. 2010). During IPT, these vulnerable humans are given a full curative antimalarial medication dose regardless of their infection status. IPT has been shown to be efficacious in reducing malaria incidence and burden in pregnant women, infants, and children (Deloron et al. 2010; Konaté et al. 2011; ter Kuile et al. 2007; Matangila et al. 2015). In particular, its use in pregnant women (via IPTp) with the drug sulfadoxine–pyrimethamine (SP) was shown to be efficacious (Deloron et al. 2010; ter Kuile et al. 2007; Matangila et al. 2015). In infants (via IPTi) and children (via IPTc), with the combination drug sulfadoxine–pyrimethamine plus amodiaquine (SP $+$ AQ), it was shown to be efficacious in reducing malaria incidence and burden (Konaté et al. 2011; Matangila et al. 2015), with significant protection for children sleeping under insecticide-treated bednets (ITNs) (Konaté et al. 2011; Matangila et al. 2015).

Although IPT (IPTp, IPTi, IPTc) as a malaria control strategy has been shown to have positive impact in averting disease deaths in IPT-treated individuals, it faces challenges due to the emergence of resistance to the drugs used for IPT treatment (Deloron et al. 2010; Gosling et al. 2010). Thus, understanding the interacting relationship between IPT use as a control strategy and the emergence and rate of spread of drug resistance is important. Models have shown the benefits to individuals in the use of IPT (Ross et al. 2008), with decreased benefits when applied inappropriately, e.g., when highly resistant strains are circulating (Ross et al. 2011). Previous modeling studies have also shown that IPTi/IPTc is likely to accelerate drug resistance spread in some situations (Ãguas et al. 2009; Alexander et al. 2007; O’Meara et al. 2006; Teboh-Ewungkem et al. 2014; Teboh-Ewungkem 2015). Teboh-Ewungkem (2015) found that while treatment of symptomatic infections is the main driver for drug resistance, IPT can increase drug-resistant malaria, particularly when a long half-life drug such as SP is used. The IPT treatment schedule can also affect the intensity of acceleration, with a critical threshold above which drug-resistant invasion is certain.

The models used to examine the role of IPT in drug resistance did not consider the direct benefits of IPT in deaths (and/or cases) averted (O’Meara et al. 2006; Teboh-Ewungkem et al. 2014; Teboh-Ewungkem 2015). In order to better understand the trade-off between deaths averted and increasing drug resistance, we adapted the Teboh-Ewungkem (2015) model to include age structure, death due to disease, and high or low transmission regions with year-round transmission. This allowed us to quantify the relative impact of IPT and inform strategies for using IPT that will maximize number of deaths averted while minimizing resistance. In particular, we considered the following quantities of interest: number of deaths averted by IPT, ratio of sensitive to resistant strains in the population across time, total number of malaria deaths, basic reproduction number and invasion reproduction number. Our goals were to (1) determine the critical level of IPT treatment that would minimize the spread of drug resistance and maximize the positive impact in lives saved; (2) determine the role of IPT in saving lives and potentially facilitating drug resistance for low and high transmission regions; and (3) understand the relative roles of symptomatic treatment and IPT in the establishment of drug-resistant strains of malaria while also considering partial resistance. In order to explicitly consider the sustainability of particular approaches, we modeled our time-varying quantities of interest for 1, 5, and 10 years. Our model differs from that of O’Meara et al. (2006) and Teboh-Ewungkem et al. (2014) in that the transmission dynamics of the vector population are explicitly modeled as well as age structure for the human hosts. The model explicitly accounted for humans with different levels of immunity as well as incorporated the dynamics of the resistant malaria strain.

The paper is divided as follows: Sect. 2 describes the model, giving the associated variables and parameters, while Sect. 3 gives a detailed analysis of the disease-free, non-trivial boundary, and endemic equilibria of the model. In Sect. 4, we present the model results and associated figures, with a parameter sensitivity analyses carried out in Sect. 5. Section 6 then gives a discussion and conclusion. We found that although IPT treatment can increase the levels and timing of resistant strain invasion, treatment of symptomatic individuals plays a much larger role in promoting resistance under our assumptions and parameter values. We also found that the prevalence of the resistant strain is highly sensitive to the half-life of the drug being administered. Successful establishment of the resistant strain is more likely when the drug being used for IPT and treatment has a long half-life. Finally, in the scenario where the symptomatic treatment drug has a short half-life and low or little resistance to the treatment drug is present in the circulating malaria strains, then using SP as an IPT drug in high transmission regions will result in many lives saved without significantly increasing resistance levels. It should be noted, however, that if strains with high resistance to the symptomatic treatment drug and the IPT drug emerge, then IPT could drive higher resistance proportions and result in an increase in number of deaths. Therefore, close monitoring of resistant strains is suggested by our model when IPT is in use.

## The Mathematical Model

### Model Formulation and Description

**Fig. 1:**
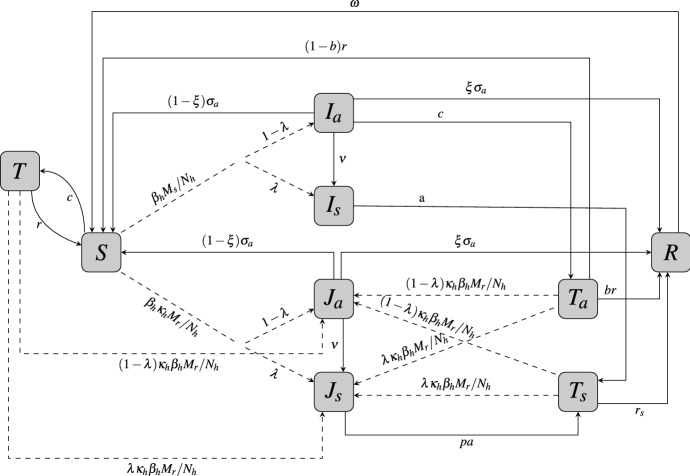
Transfer diagram for human infection within the naive-immune population. Dashed lines represent parasite transmission via infected mosquitoes. *I* infections are with sensitive strains and *J* with resistant strains of malaria with subscripts *a* and *s* representing asymptomatic and symptomatic cases. *T* and $T_\mathrm{a}$ are susceptible and asymptomatic individuals, respectively, that received IPT, while $T_\mathrm{s}$ is individuals receiving treatment for a symptomatic case. *S* is fully susceptible, and *R* is temporarily immune

**Fig. 2:**
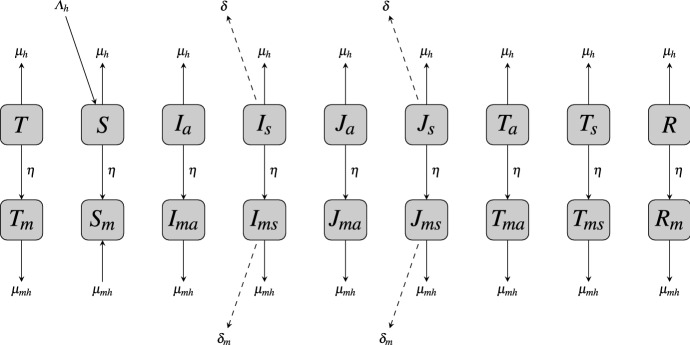
Transfer diagram between the naive-immune juvenile human population and the mature human population. Dashed lines represent disease-induced mortality. An average time of $1/\eta$ is spent in the naive-immune class

**Fig. 3:**
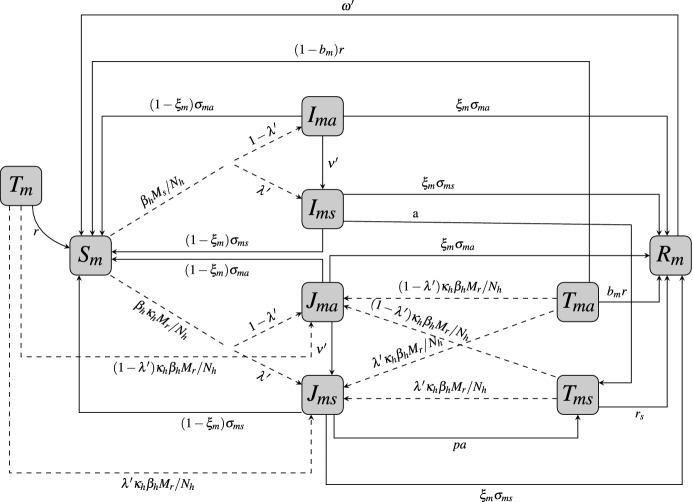
Transfer diagram for human infection within the mature population. Dashed lines represent parasite transmission via infected mosquitoes. $T_\mathrm{ma}$ and $T_\mathrm{m}$ are holding compartments for individuals that mature while in an IPT treatment class (so drug is still circulating in their system). The subscript m indicates immune-mature individuals, but all other notation is the same as in Fig. 1

The model developed here extends the IPT model in Teboh-Ewungkem (2015) by explicitly including age structure and disease-induced mortalities in the human populations. See Figs. 1, 2, and 3 for the updated model flowcharts and Tables 1, 2, and 3 for the definitions of the variables and parameters. The new model equations are described by (1a)–(1j), (2a)–(2j), and (3a)–(3c) for the human population and (4b)–(4c) and (5a) for the mosquito population.

The equations are a system of nonlinear ordinary differential equations age-structured variable-population model with IPT usage incorporated. The human population is split into two age groups based on their status of acquired immunity: juveniles, with a naive or no clinical acquired immunity, and mature humans, who have a higher level of clinical immunity to malaria, due to frequent exposure to the parasites (Klein et al. 2008; Teboh-Ewungkem et al. 2014; Woldegerima et al. 2018). By clinical immunity, we mean the gradual acquisition of parasite-exposed-primed immune response enabling an individual to be symptom-free even though they might have the transmissible forms of the parasites in their blood stream (Cohen et al. 1961). Thus, mature humans, those considered to have a more developed acquired immunity usually, do not feel ill from the malaria parasite infection (Klein et al. 2008; Teboh-Ewungkem et al. 2014; Woldegerima et al. 2018), which can be associated with less severe malaria symptoms. Thus, the rates of antimalarial drug use among these mature individuals are considered to be lower (Klein et al. 2008; Teboh-Ewungkem et al. 2014). They will not be administered IPT. On the other hand, juveniles, the infants, and children, with naive acquired immunity, are those receiving IPTi or IPTc, respectively. Typically, the juvenile population will consist of the 0–5-years-old age group and $>\,5$ years old the mature group. However, this age group can be extended or made shorter depending on the transmission intensity of the region (low or high) and/or whether the region has stable or unstable transmission with transmission either occurring all year round (holoendemicity) or intermittently with periods of intense transmission (hyperendemicity) (Hay et al. 2008). Note the simplifying assumptions on immunity development taken by the model. While more complex immunity development patterns may be important, for simplicity the model only considers two levels of immunity: naive and mature.

In the model, the juvenile and mature human populations are each subdivided into mutually exclusive compartments categorized by malaria strain-type disease infection or treatment status. Henceforth, we will refer to IPTi and IPTc as just IPT. A human, juvenile of mature, upon contact with an infectious mosquito may be successfully infected with a sensitive malaria parasite strain or a resistant parasite strain. The infected individual may show symptoms, considered to be symptomatic (identified by the subscript *a*), or may not show symptoms, considered to be asymptomatic. Symptomatic individuals, mature or juvenile, receive treatment. Thus, the compartments for the juveniles at any time *t* are: susceptible juveniles (denoted by *S*), symptomatic juveniles infected with the sensitive strain ($I_\mathrm{s}$) or the resistant strain ($J_\mathrm{s}$), asymptomatic juveniles infected with the sensitive strain ($I_\mathrm{a}$) or the resistant strain ($J_\mathrm{a}$), susceptible juveniles who have received IPT (*T*), asymptomatic infected juveniles who received IPT ($T_\mathrm{a}$), treated symptomatic infected juveniles ($T_\mathrm{s}$), and the temporarily immune juveniles (*R*), see Fig. 1. As juveniles age, they join a corresponding mature human population class (see Fig. 2). Denoting the corresponding mature human classes by the subscript *m*, the compartments for the mature human population at time *t* are: susceptible individuals ($S_\mathrm{m}$), symptomatic infected with the sensitive strain ($I_\mathrm{ms}$) or the resistant strain ($J_\mathrm{ms}$), asymptomatic individuals infected with the sensitive strain ($I_\mathrm{ma}$) or the resistant strain ($J_\mathrm{ma}$), uninfected juveniles who received IPT and aged, aging into the mature class ($T_\mathrm{m}$), infected asymptomatic juveniles who received IPT and aged, aging into the mature class ($T_\mathrm{ma}$), treated symptomatic infected humans ($T_\mathrm{ms}$), and temporarily immune humans ($R_\mathrm{m}$), see Fig. 3. Additionally, at any time *t*, there are a number $S_v$ (susceptible mosquitoes) and *M* (infectious mosquitoes) that define the mosquito classes. The *M* mosquitoes are further subdivided into subclasses $M_\mathrm{r}$ and $M_\mathrm{s}$ which determines the type of parasite they are infected with, sensitive or resistant. Thus, the total mosquito population at time *t*, denoted by $N_v$, is $N_v=S_v + M_\mathrm{r} + M_\mathrm{s}$. A detailed description of all the variable classes is given in Table 1.

Additionally, contact between an infected mosquito and a susceptible human may lead to the human being infected with the sensitive parasite strain, identified by the variable *I*, if the bite came from a $M_\mathrm{s}$-type mosquito, or a resistant parasite strain, identified by the variable *J*, if the bite came from a $M_\mathrm{r}$-type mosquito. It is possible for the strains to differ in fitness, noted by $\kappa _\mathrm{h}$, the fitness difference for the resistant strain. The factor $\kappa _\mathrm{h}$ multiplies the transmission terms for individuals (whether mosquito or human) infected with the resistant strain. We assume $0\le \kappa _\mathrm{h} \le 1$. In summary, an infectious human, naive-immune and mature-immune, may be symptomatic and infected with the sensitive parasite strain (classes $I_\mathrm{s}$ and $I_\mathrm{ms}$), or the resistant parasite strain (classes $J_\mathrm{s}$ and $J_\mathrm{ms}$), or asymptomatic and infected with the sensitive parasite strain (classes $I_\mathrm{a}$ and $I_\mathrm{ma}$), or the resistant parasite strain (classes $J_\mathrm{a}$ and $J_\mathrm{ma}$). We note that we do not consider coinfection in our model. Thus, any individual coinfected with the sensitive or resistant parasite strain is considered a resistant infectious human.

**Table 1:** State variables and their descriptions

Variable	Description of variable
$S_{v}$	Number of susceptible mosquitoes
$M_\mathrm{s}$	Number of mosquitoes infected with the sensitive strain
$M_\mathrm{r}$	Number of mosquitoes infected with the resistant strain
*S*	Number of susceptible juveniles
$I_\mathrm{s}$	Number of symptomatic infected juveniles infected with the sensitive parasite strain
$I_\mathrm{a}$	Number of asymptomatic infected juveniles infected with the sensitive parasite strain.
$J_\mathrm{s}$	Number of symptomatic infected juveniles infected with the resistant parasite strain
$J_\mathrm{a}$	Number of asymptomatic infected juveniles infected with the resistant parasite strain
$T_\mathrm{s}$	Number of symptomatic infected juveniles who are treated due to their symptoms
*T*	Number of susceptible juveniles who have received IPT treatment.
$T_\mathrm{a}$	Number of asymptomatic infected juveniles who have received IPT treatment
*R*	Number of infected juveniles who clear their parasite either naturally or via treatment and develop temporary immunity
$S_\mathrm{m}$	Number of susceptible mature humans
$I_\mathrm{ms}$	Number of symptomatic infectious mature humans infected with the sensitive strain
$I_\mathrm{ma}$	Number of asymptomatic infected mature humans infected with sensitive strain
$J_\mathrm{ms}$	Number of symptomatic infected mature humans infected with the resistant strain
$J_\mathrm{ma}$	Number of asymptomatic infected mature humans infected with the resistant strain
$T_\mathrm{m}$	Number of susceptible juveniles who had received IPT and aged prior to their drug levels declining to the levels that rendered them susceptible
$T_\mathrm{ma}$	Number of asymptomatic juveniles who had received IPT and aged prior to their drug levels declining to the levels that rendered them temporary immune or susceptible
$T_\mathrm{ms}$	Number of mature humans who receive treatment due to their symptomatic infection
$R_\mathrm{m}$	Number of infected mature humans who clear their parasite either naturally or via treatment and develop temporary immunity
$N_{c}$	Total number of juvenile population
$N_\mathrm{m}$	Total number of mature human population
$N_\mathrm{h}$	Total human population

In our model, we assume that only the symptomatic humans (juveniles or mature) will seek treatment, with the assumption that symptomatic naive-immune individuals clear their symptomatic parasite infections only via treatment (due to their poor or less developed immune state), else they will die from the infection. On the other hand, in addition to treatment methods, symptomatic mature-immune individuals can also clear their parasite naturally because of their developed immune response. Symptomatic individuals who do not clear their infections (ether via treatment and/or naturally) can die from the disease, at the rate $\delta$, for naive-immune individuals and $\delta _\mathrm{m}$, for mature-immune individuals. Typically, $\delta > \delta _\mathrm{m}$, Desai et al. (2014), with up to a 10 folds difference reported in some regions.

The baseline drugs considered for treatment of symptomatic malaria infections are the WHO recommended combination therapy drugs such as artemether–lumefantrine (also called coartem, referred henceforth as the AL drug) or other approved artemisinin-based combination therapy drugs (ACT drugs) (World Health Organization 2015a, b). However, we will investigate the impact of a long half-life drug such as sulphadoxine–pyrimethamine (SP) as a treatment drug for symptoms. If a symptomatic naive-immune (respectively, mature-immune) individual, infected with the sensitive parasite strain, receives treatment, they move to the treatment class $T_\mathrm{s}$ (respectively, $T_\mathrm{ms}$), at rate *a*. 1 / *a* is the average time from the onset of treatment to the clearance of the sensitive parasite. If the individual (naive-immune and mature-immune) is infected with the resistant parasite strain, we assume that the drug is ineffective against the resistant parasite. Thus, such infectious humans, type $J_\mathrm{s}$ and $J_\mathrm{ms}$ individuals, move to their corresponding treatment classes, class $T_\mathrm{s}$, respectively, $T_\mathrm{ms}$, at rate *pa*, where *p* measures the efficacy of the drug against a resistant infection. We note that *p* can account for full resistance (in which case $p=0$) or partial resistance (in which case $p>0$). In addition, mature-immune symptomatic humans can also clear their infection naturally at rate $\sigma _\mathrm{ms}$, with a proportion $\xi _\mathrm{m}$ developing temporal immunity to join the temporal immune class *R*, and the remainder $1-\xi _\mathrm{ms}$ joining the susceptible mature human class.

Asymptomatic infectious individuals (naive-immune and mature-immune) do not seek treatment because they do not show symptoms even though considered to be clinically sick and infectious. However, these individuals can clear their parasitic infections naturally at rate $\sigma _\mathrm{a}$ and $\sigma _\mathrm{ma}$, respectively, with a proportion $\xi$ and $\xi _\mathrm{m}$, respectively, developing temporal immunity to join the temporal immune classes *R* and $R_\mathrm{m}$. The remainder, $1-\xi$ and $1-\xi _\mathrm{m}$, instead join the susceptible naive immune (*S*) and mature human ($S_\mathrm{m}$) classes. We also assumed that asymptomatic infectious humans (naive-immune and mature-immune) can develop symptoms at rates $\nu$ and $\nu ^\prime$, respectively.

As a preventative measure, both susceptible and asymptomatic naive-immune individuals receive intermittent preventive treatment (IPT), as in O’Meara et al. (2006), Teboh-Ewungkem et al. (2014), Teboh-Ewungkem (2015). IPT is administered at a constant per capita rate *c*, where 1 / *c* is the average time between IPT treatments. We adopt the WHO recommended drug for IPT treatment, sulphadoxine–pyrimethamine (SP), a long half-life drug (Teboh-Ewungkem et al. 2014; Teboh-Ewungkem 2015; World Health Organization 2015a, b), as the baseline IPT treatment drug. Naive-immune juveniles who receive IPT will move to the IPT-treated class *T*, if the IPT was administered to a susceptible juvenile, and to $T_\mathrm{a}$, if the IPT was administered to an asymptomatic infectious juvenile.

All treated individuals, mature or naive-immune, are assumed to have drugs at therapeutic levels in their system that can clear sensitive parasites, regardless of whether the treatment was due to a symptomatic infection (classes $T_\mathrm{s}$ and $T_\mathrm{ms}$ individuals), or IPT (classes *T* and $T_\mathrm{a}$). As the drug concentration in these treated individuals declines, the individuals may either join the temporarily immune or the susceptible class. In particular, as the drug concentration in individuals treated due to a symptomatic infection declines (at rate $r_\mathrm{s}$), the individuals join the temporary immune class, with $T_\mathrm{s}$ moving to *R* and $T_\mathrm{ms}$ moving to $R_\mathrm{m}$. The rate $r_\mathrm{s}$ depends on the half-life of the drug used for treatment, with $1/r_\mathrm{s}$ the time in days the treatment drug reaches levels that do not have therapeutic effects on a sensitive parasite infection. We have assumed here that an immune response is triggered as a result of malaria symptoms, hence the development of temporary immunity. For individuals who receive IPT, the rate of decline of the drug is *r*. If the IPT was administered to a susceptible naive-immune individual, generating a type *T* naive-immune juvenile, the individual will move to the susceptible class *S*, as their drug concentration declines. However, if the IPT was administered to an asymptomatic infectious naive-immune juvenile, generating a type $T_\mathrm{a}$ naive-immune juvenile, a proportion *b* of these juveniles will move to class *R*, while the remainder $1-b$, will join class *S*. The separation is justified in that an asymptomatic infection is as a result of some naive level of temporal immunity bolstered by the IPT drug. Here, 1 / *r* is the time in days the IPT drug is at levels that do not have therapeutic effects on a sensitive parasite. Temporarily immune individuals (in classes *R* and $R_\mathrm{m}$) lose their temporary immune status to join the susceptible class at a rate $\omega$ for naive-immune and $\omega ^{\prime }$ for mature-immune individuals.

We assume in our model that after age 5 (could be shorter for a stable high transmission region), a naive-immune juvenile matures to join an equivalent corresponding mature class. This maturation happens at a constant per capita rate of $\eta$ with $1/\eta$ the age considered for the naive-immune individual to have developed a reasonable immune response due to repeated re-exposure to the malaria parasite. For naive-immune treated individuals who received IPT, we assume that if they mature while receiving IPT, they move into a temporary IPT treatment compartment in the mature group represented by classes $T_\mathrm{m}$ and $T_\mathrm{ma}$. When the drug concentration of these individuals declines at the stated rate *r*, they either join the susceptible mature or the temporary immune mature classes, with $T_\mathrm{m}$ individuals moving to class $S_\mathrm{m}$ and a proportion $b_\mathrm{m}$ of the $T_\mathrm{ma}$ individuals moving to class $R_\mathrm{m}$ while the remaining proportion, $1-b_\mathrm{m}$, moves to class $S_\mathrm{m}$. Since no mature-immune humans receive IPT, there is no movement of mature-immune individuals into class $T_\mathrm{m}$ or $T_\mathrm{ma}$.

Here, we assume that all recruitment via births occur at a constant rate $\varLambda _\mathrm{h}$ into the susceptible naive-immune class, and that natural death can occur from all compartments at a constant per capita death rate of $\mu _\mathrm{h}$ for the naive-immune individuals or $\mu _{mh}$ for the mature-immune individuals. Figure 2 illustrates movement due to maturation from the naive-immune compartments into the parallel mature-immune compartments, indicating where there is disease-induced deaths, natural death, and recruitment. The equations governing the human disease dynamics are given in (1a)–(1j) for the naive-immune human population, (2a)–(2j), for the mature-immune human population, and (3a)–(3c) for the subtotal naive-immune, subtotal mature-immune, and the total human populations.

When a susceptible mosquito feeds, successfully taking blood from an infectious human, the mosquito may acquire the malaria parasite from the human at rate $\beta _{v}$, moving to either the $M_\mathrm{s}$ or $M_\mathrm{r}$ class. If the blood meal was from a human infected with the sensitive parasite strain, then the mosquito, with a successful infection with the sensitive parasites, will become a type $M_\mathrm{s}$ mosquito. If, on the other hand, the blood meal was from a human infected with the resistant parasite strain, then a successful infection with resistant parasites will render the mosquito a type $M_\mathrm{r}$ mosquito. Here, we also assume that the transmission success to mosquitoes by humans infected with the resistant parasite is less than that from humans infected with the sensitive parasite. Thus, the transmission rate of resistant parasites to susceptible mosquitoes is $\kappa _{v}\beta _{v}$, where $0< \kappa _{v} < 1$ is the transmission reduction factor. We further assume that a mosquito cannot be coinfected, that is, if a mosquito is infected with a particular strain of malaria, the mosquito will not acquire nor successfully transmit a second distinct strain of malaria. Thus, there is no movement between the $M_\mathrm{s}$ and $M_\mathrm{r}$ compartments; once a mosquito is infected, it remains so until it dies; and natural death occurs from each mosquito compartment at rate $\mu _{v}$. The equations governing the mosquito dynamics are given in (4a)–(4c), with the total mosquito population modeled by (5a).

1a $$\begin{aligned} \frac{\mathrm{d}S}{\mathrm{d}t}&= \varLambda _\mathrm{h} - \mu _\mathrm{h} S- \beta _\mathrm{h}(M_\mathrm{s} +\kappa _\mathrm{h} M_\mathrm{r})S/N_\mathrm{h} - c S + (1-\xi )\sigma _\mathrm{a} (I_\mathrm{a} + J_\mathrm{a}) \end{aligned}$$

1b $$\begin{aligned}&\quad + (1-b) r T_\mathrm{a}+ r T + \omega R-\eta S, \end{aligned}$$

1c $$\begin{aligned} \frac{\mathrm{d}I_\mathrm{s}}{\mathrm{d}t}&= \lambda \beta _\mathrm{h} M_\mathrm{s} S/N_\mathrm{h} + \nu I_\mathrm{a} - (a + \mu _\mathrm{h} + \eta + \delta ) I_\mathrm{s}, \end{aligned}$$

1d $$\begin{aligned} \frac{\mathrm{d}I_\mathrm{a}}{\mathrm{d}t}&= (1-\lambda )\beta _\mathrm{h} M_\mathrm{s} S/N_\mathrm{h} - (c + \nu + \sigma _\mathrm{a} + \mu _\mathrm{h} + \eta ) I_\mathrm{a}, \end{aligned}$$

1e $$\begin{aligned} \frac{\mathrm{d}J_\mathrm{s}}{\mathrm{d}t}&= \lambda \kappa _\mathrm{h} \beta _\mathrm{h} M_\mathrm{r}[S + T_\mathrm{s} + T + T_\mathrm{a}]/N_\mathrm{h} + \nu J_\mathrm{a} - (pa + \mu _\mathrm{h} + \eta + \delta )J_\mathrm{s}, \end{aligned}$$

1f $$\begin{aligned} \frac{\mathrm{d}J_\mathrm{a}}{\mathrm{d}t}&= (1-\lambda ) \kappa _\mathrm{h} \beta _\mathrm{h} M_\mathrm{r}[S + T_\mathrm{s} + T + T_\mathrm{a}]/N_\mathrm{h} - (\sigma _\mathrm{a} + \nu + \mu _\mathrm{h} + \eta ) J_\mathrm{a} , \end{aligned}$$

1g $$\begin{aligned} \frac{\mathrm{d}T_\mathrm{s}}{\mathrm{d}t}&= a I_\mathrm{s} + pa J_\mathrm{s}- r_\mathrm{s} T_\mathrm{s} - \kappa _\mathrm{h} \beta _\mathrm{h} M_\mathrm{r} T_\mathrm{s}/N_\mathrm{h} - (\mu _\mathrm{h} + \eta ) T_\mathrm{s}, \end{aligned}$$

1h $$\begin{aligned} \frac{\mathrm{d}T}{\mathrm{d}t}&= c S - r T - \kappa _\mathrm{h} \beta _\mathrm{h} T M_\mathrm{r}/N_\mathrm{h} - (\mu _\mathrm{h} + \eta ) T, \end{aligned}$$

1i $$\begin{aligned} \frac{\mathrm{d}T_\mathrm{a}}{\mathrm{d}t}&= c I_\mathrm{a} - r T_\mathrm{a} - \kappa _\mathrm{h} \beta _\mathrm{h} T_\mathrm{a} M_\mathrm{r}/N_\mathrm{h} - \mu _\mathrm{h} T_\mathrm{a} - \eta T_\mathrm{a}, \end{aligned}$$

1j $$\begin{aligned} \frac{\mathrm{d}R}{\mathrm{d}t}&= r_\mathrm{s} T_\mathrm{s} + b r T_\mathrm{a} + \xi \sigma _\mathrm{a} (I_\mathrm{a} + J_\mathrm{a}) - (\omega + \mu _\mathrm{h} + \eta ) R,, \end{aligned}$$

2a $$\begin{aligned} \frac{\mathrm{d}S_\mathrm{m}}{\mathrm{d}t}&= \eta S - \mu _{mh} S_\mathrm{m} - \beta _\mathrm{h}(M_\mathrm{s} + \kappa _\mathrm{h} M_\mathrm{r})S_\mathrm{m}/N_\mathrm{h} + (1-\xi _\mathrm{m})\sigma _\mathrm{ma} (I_{ma} + J_\mathrm{ma}) \end{aligned}$$

2b $$\begin{aligned}&\quad +(1-\xi _\mathrm{m})\sigma _\mathrm{ms} (I_{ms} + J_\mathrm{ms})+ \omega ^{\prime } R_\mathrm{m} + r T_\mathrm{m} +(1-b_\mathrm{m}) r T_\mathrm{ma}, \end{aligned}$$

2c $$\begin{aligned} \frac{\mathrm{d}I_\mathrm{ms}}{\mathrm{d}t}&= \eta I_\mathrm{s} + \lambda ^{\prime } \beta _\mathrm{h} M_\mathrm{s} S_\mathrm{m}/N_\mathrm{h} + \nu ^{\prime } I_\mathrm{ma} - (a + \mu _{mh} + \delta _\mathrm{m} + \sigma _\mathrm{ms}) I_{ms}, \end{aligned}$$

2d $$\begin{aligned} \frac{\mathrm{d}I_\mathrm{ma}}{\mathrm{d}t}&= \eta I_\mathrm{a} + (1-\lambda ^{\prime })\beta _\mathrm{h} M_\mathrm{s} S_\mathrm{m}/N_\mathrm{h} - (\sigma _\mathrm{ma} + \nu ^{\prime } +\mu _{mh}) I_\mathrm{ma} , \end{aligned}$$

2e $$\begin{aligned} \frac{\mathrm{d}J_\mathrm{ms}}{\mathrm{d}t}&= \eta J_\mathrm{s} + \lambda ^{\prime } \kappa _\mathrm{h} \beta _\mathrm{h} M_\mathrm{r}[S_\mathrm{m}+ T_\mathrm{ms} + T_\mathrm{m} + T_\mathrm{ma}]/N_\mathrm{h} + \nu ' J_\mathrm{ma} \nonumber \\&\quad - (pa +\sigma _\mathrm{ms} + \mu _{mh} + \delta _\mathrm{m})J_\mathrm{ms}, \end{aligned}$$

2f $$\begin{aligned} \frac{\mathrm{d}J_\mathrm{ma}}{\mathrm{d}t}&= \eta J_\mathrm{a}+ (1-\lambda ^{\prime })\kappa _\mathrm{h} \beta _\mathrm{h} M_\mathrm{r}[S_\mathrm{m} + T_\mathrm{ms} + T_\mathrm{m} + T_\mathrm{ma}]/N_\mathrm{h} \nonumber \\&\quad - (\sigma _\mathrm{ma} + \nu ^{\prime } + \mu _{mh})J_\mathrm{ma}, \end{aligned}$$

2g $$\begin{aligned} \frac{\mathrm{d}T_\mathrm{ms}}{\mathrm{d}t}&= \eta T_\mathrm{s} + a I_\mathrm{ms} + pa J_\mathrm{ms}- \kappa _\mathrm{h} \beta _\mathrm{h} M_\mathrm{r} T_\mathrm{ms}/N_\mathrm{h} - (\mu _{mh} + r_\mathrm{s}) T_\mathrm{ms}, \end{aligned}$$

2h $$\begin{aligned} \frac{\mathrm{d}T_\mathrm{m}}{\mathrm{d}t}&= \eta T - \kappa _\mathrm{h} \beta _\mathrm{h} T_\mathrm{m} M_\mathrm{r}/N_\mathrm{h} - (\mu _{mh} + r) T_\mathrm{m}, \end{aligned}$$

2i $$\begin{aligned} \frac{\mathrm{d}T_\mathrm{ma}}{\mathrm{d}t}&= \eta T_\mathrm{a} - \kappa _\mathrm{h} \beta _\mathrm{h} T_\mathrm{ma} M_\mathrm{r}/N_\mathrm{h} - (\mu _{mh} + r) T_\mathrm{ma}, \end{aligned}$$

2j $$\begin{aligned} \frac{\mathrm{d}R_\mathrm{m}}{\mathrm{d}t}&= \eta R + r_\mathrm{s} T_\mathrm{ms} + b_\mathrm{m} r T_\mathrm{ma} + \xi _\mathrm{m} \sigma _\mathrm{ma} (I_{ma}+ J_\mathrm{ma} ) + \xi _\mathrm{m} \sigma _\mathrm{ms}(I_{ms}+J_\mathrm{ms})\nonumber \\&\quad -\omega ^{\prime } R_\mathrm{m} - \mu _{mh} R_\mathrm{m}, \end{aligned}$$

In our model, the total juvenile population is $N_{c}=S + I_\mathrm{s}+I_\mathrm{a} + J_\mathrm{s}+ J_\mathrm{a} + T + T_\mathrm{s} + T_\mathrm{a} + R$, the total mature population is $N_\mathrm{m}=S_\mathrm{m} + I_\mathrm{ms}+I_\mathrm{ma} + J_\mathrm{ms}+ J_\mathrm{ma} + T_\mathrm{m} + T_\mathrm{ms} + T_\mathrm{ma} + R_\mathrm{m}$, so that the total human population $N_\mathrm{h} = N_c + N_\mathrm{m}$. The equations that model the $N_{c}$, $N_\mathrm{m}$, and $N_\mathrm{h}$ populations are:

3a $$\begin{aligned} \frac{\mathrm{d}N_c}{\mathrm{d}t}&= \varLambda _\mathrm{h} - \eta N_c - \mu _\mathrm{h} N_c - \delta (I_\mathrm{s} +J_\mathrm{s}), \end{aligned}$$

3b $$\begin{aligned} \frac{\mathrm{d}N_\mathrm{m}}{\mathrm{d}t}&= \eta N_c - \mu _{mh} N_\mathrm{m} - \delta _\mathrm{m} (I_\mathrm{ms} +J_{ms}), \end{aligned}$$

3c $$\begin{aligned} \frac{\mathrm{d}N_\mathrm{h}}{\mathrm{d}t}&= \varLambda _\mathrm{h} - \mu _\mathrm{h} N_c - \mu _{mh} N_\mathrm{m} - \delta (I_\mathrm{s} +J_\mathrm{s}) - \delta _\mathrm{m} (I_\mathrm{ms} +J_{ms}). \end{aligned}$$

The total human population has a disease-free carrying capacity of $N_\mathrm{h}^*=\varLambda _\mathrm{h} /(\psi \mu _\mathrm{h}+(1-\psi )\mu _{mh})$, where $\psi N_\mathrm{h} = N_c$ is the total naive-immune human population, and $(1-\psi ) N_\mathrm{h}^* = N_\mathrm{m}^*$ is the total mature-immune human population and $N_c^* = \varLambda _\mathrm{h}/(\nu + \mu _\mathrm{h})$ and $N_\mathrm{m}^* = \eta N_c/\mu _{mh}$ are the equilibria of the juvenile and mature populations without death from malaria. Thus, $\psi$ gives the ratios of naive-immune to the total human populations so that $N_c^* + N_\mathrm{m}^* = N_\mathrm{h}^*$, the total human population.

The equations that govern the mosquito dynamics are

4a $$\begin{aligned} \frac{\mathrm{d}S_v}{\mathrm{d}t}&= \varLambda _v - \beta _v \left[ I_\mathrm{a}+I_\mathrm{s}+ I_\mathrm{ma} + I_\mathrm{ms} + \kappa _{v} (J_\mathrm{a} + J_\mathrm{s}+ J_\mathrm{ma} + J_\mathrm{ms}) \right] S_v/N_\mathrm{h} -\mu _v S_v, \end{aligned}$$

4b $$\begin{aligned} \frac{\mathrm{d}M_\mathrm{s}}{\mathrm{d}t}&= \beta _v (I_\mathrm{a} + I_\mathrm{s} + I_\mathrm{ma} + I_\mathrm{ms})S_v/N_\mathrm{h} - \mu _v M_\mathrm{s}, \end{aligned}$$

4c $$\begin{aligned} \frac{\mathrm{d}M_\mathrm{r}}{\mathrm{d}t}&=\kappa _{v} \beta _v (J_\mathrm{a} + J_\mathrm{s}+ J_\mathrm{ma} + J_\mathrm{ms}) S_v/N_\mathrm{h} - \mu _v M_\mathrm{r}, \end{aligned}$$

where the total mosquito population is $N_{v}=S_v + M_\mathrm{s} + M_\mathrm{r}$ and is modeled by the equation

5a $$\begin{aligned} \frac{\mathrm{d}N_v}{\mathrm{d}t}&= \varLambda _v - \mu _v N_v. \end{aligned}$$

The total mosquito population is also non-constant, with a disease-free carrying capacity of $\varLambda _v /\mu _v$.

We remark that in our model discussions, we consider the number of bites per day a human gets to be limited by mosquito density, not human density, i.e., every mosquito gets to bite as often as they desire. Therefore, the total number of bites per day is defined as (the number of bites desired per day by a mosquito) $\times$ (total number of mosquitoes) $= \alpha N_v$, where $N_v$ is the total number of mosquitoes and $\alpha$ is the number of bites per mosquito per day. Thus, the number of bites per person per day is $\alpha N_v / N_\mathrm{h}$, where $N_\mathrm{h}$ is the total number of humans. See (Chitnis et al. 2006) for a discussion of alternative biting rates as the vector-to-host ratio becomes either very low or very high. Thus, $\beta _\mathrm{h}$ is then the product of the mosquito biting rate ($\alpha$, or number of bites on humans per mosquito per day) times the probability that transmission occurs if the bite is from an infectious mosquito (represented by $\beta _{hv}$). On the other hand, $\beta _v$ is the product of the mosquito biting rate times the probability that transmission occurs if the bite is on an infectious individual (represented by $\beta _{vh}$).

Table 1 summarizes the state variable descriptions. All parameters, as defined in Tables 2 and 3, are non-negative. Details about their interpretation and values will be presented in Sect. 2.2. With nonnegative initial conditions, it can be verified that the solutions to the model equations remain non-negative.

**Table 2:** Descriptions and dimensions for parameters related to the natural transmission cycle

Parameter	Description	Dimension
$\varLambda _\mathrm{h}$	Total human birth rate	humans $T^{-1}$
$\varLambda _{v}$	Total mosquito birth rate	mosquitoes $T^{-1}$
$\mu _\mathrm{mh}$	Per capita death rate of mature humans	$T^{-1}$
$\mu _\mathrm{h}$	Per capita death rate of juveniles	$T^{-1}$
$\delta _\mathrm{m}$	Malaria disease-induced mortality rate for mature humans	$T^{-1}$
$\delta$	Malaria disease-induced mortality rate for juveniles	$T^{-1}$
$\mu _{v}$	Natural mosquito death rate	$T^{-1}$
$\eta$	Rate of aging, i.e., rate at which juveniles become mature humans and no longer receive IPT	$T^{-1}$
$\beta _\mathrm{h}$	Transmission rate of sensitive parasites from mosquitoes to humans ($\alpha \beta _{hv}$)	$\hbox {mosquito}^{-1} T^{-1}$
$\beta _{v}$	Transmission rate of sensitive parasites from humans to mosquitoes ($\alpha \beta _{vh}$)	$\hbox {human}^{-1}T^{-1}$
$\kappa _\mathrm{h}$	Reduction factor of human transmission rate by the resistant parasite strain	1
$\kappa _{v}$	Reduction factor of mosquito transmission rate by the resistant parasite strain	1
$\lambda$	Fraction of juveniles who become symptomatic upon infection	1
$\lambda ^\prime$	Fraction of matures who become symptomatic upon infection	1
$\omega$	Rate of loss of temporary immunity in juveniles	$T^{-1}$
$\omega ^\prime$	Rate of loss of temporary immunity in mature adults	$T^{-1}$
$\lambda$	Fraction of juveniles who become symptomatic upon infection	1
$\lambda ^\prime$	Fraction of matures who become symptomatic upon infection	1
$\nu$	Rate at which juveniles progress from asymptomatic to symptomatic infections	$T^{-1}$
$\nu ^\prime$	Rate at which mature humans progress from asymptomatic to symptomatic infections	$T^{-1}$
$\sigma _\mathrm{s}$	Rate of naturally clearing a symptomatic infection for juveniles	$T^{-1}$
$\sigma _\mathrm{a}$	Rate of naturally clearing an asymptomatic infection for juveniles	$T^{-1}$
$\sigma _\mathrm{ms}$	Rate of naturally clearing a symptomatic infection for matures	$T^{-1}$
$\sigma _\mathrm{ma}$	Rate of naturally clearing an asymptomatic infection for matures	$T^{-1}$
$\xi$	Proportion of asymptomatic juveniles who naturally clear their infection and develop temporary immunity	1
$\xi _\mathrm{m}$	Proportion of mature humans who naturally clear their infection and develop temporary immunity	1
$\delta$	Disease-induced death rate for juveniles	$T^{-1}$
$\delta _\mathrm{m}$	Disease-induced death rate for mature humans	$T^{-1}$

**Table 3:** Descriptions and dimensions for parameters related to symptomatic treatment and IPT

Parameter	Description	Dimension
1 / *a*	Days to clear a sensitive infection after treatment	*T*
*c*	Per capita rate of IPT treatment administration	$T^{-1}$
1 / *r*	Time chemoprophylaxis lasts in IPT-treated humans	*T*
$1/r_\mathrm{s}$	Time chemoprophylaxis lasts in symptomatic treated humans	*T*
*b*	Fraction of asymptomatic infected treated juveniles who become temporarily immune protected	1
$b_\mathrm{m}$	Fraction of asymptomatic infected treated mature humans who become temporarily immune protected	1
*p*	Efficacy of drugs used to clear resistant infections	1

### Parameters

In this section, we present a discussion of the parameters used in the model. The chemoprophylaxis IPT drug considered here is sulphadoxine–pyrimethamine (SP), a drug with a long half-life (148–256 h). Drugs with long half-lives are slowly eliminated from the body compared to those with shorter half-lives, and are therefore expected to impose greater selective pressure for drug resistance than those with shorter half-lives (Babiker et al. 2009). The expectation is that drugs that persist longer in the body at sub-therapeutic levels will provide more opportunities for non-resistant (susceptible) parasites to acquire resistant traits, and for partially resistant parasites to become fully resistant. Resistance to SP, a long half-life drug, is common, while resistance to artemether–lumefantrine (AL ) or other approved artemisinin-based combination therapy drugs (ACT), short half-life drugs, has not been reported in most African countries. Typically, SP, the long half-life drug, is used for IPT, while the short half-life drugs ACT or AL are used to treat infections. ACT and AL currently work against both sensitive and resistant parasites in most regions, so are associated with values of *p* closer to 1. If resistance develops to these, then the value of *p* for treatment drugs will be closer to 0. On the other hand, SP clears sensitive parasites but not resistant parasites. Note that since short half-life drugs such as ACT and AL at therapeutic levels are effective against resistant parasites, if we consider their use as IPT drugs, then we may need to add an additional link from $J_\mathrm{a}$ to $T_\mathrm{a}$ but with much lower effectiveness. The lower effectiveness against clearance of resistant parasites comes as a result of the way IPT is administered, with long intervals between administration, allowing for opportunities for the drug to dip below therapeutic levels between treatments (Greenwood 2010). In this manuscript, we assume that asymptomatic infection by resistant parasites is untreated, since these individuals do not seek treatment and for those receiving IPT we assume a negligible impact on clearance. On the other hand, symptomatic infections by resistant parasites have higher clearance success rates if treated with an AL or ACT drug, or are partially treatable if treated with SP (this as a result of symptoms making it possible for the drug to bolster the symptom-initiated body’s natural and adaptive immune response aiding in parasite clearance.^1^

The parameters $1/r_\mathrm{s}$ and 1 / *r* give the respective average time chemoprophylaxis lasts in symptomatic treated and IPT-treated humans, respectively. These values were estimated based on reported half-lives values for antimalarial drugs. O’Meara et al. (2006) reported that for a drug with a long half-life such as sulfadoxine–pyrimethamine (SP), it takes about 52 days for the drug concentration to drop below a threshold value that it cannot clear malaria parasites, while for a drug with a short half-life, such as AL or ACT, this time period is about 6 days (Makanga and Krudsood 2009). These are the same values used in Teboh-Ewungkem (2015). For the number of IPT treatments given per person per day, *c*, we use the value $0.016\ \hbox {day}^{-1}$ as in O’Meara et al. (2006), Teboh-Ewungkem (2015). This value corresponds to IPT being given once every $60 \hbox {days}$, or 1 / *c*. Since a goal of this manuscript is to see the impact of IPT in averting disease-induced deaths, we will vary *c* to see the role frequency of IPT administration might have on the number of child disease-induced mortality and the rate of resistance spread.

The average number of days needed to clear an infection with appropriate treatment is 1 / *a*. Assuming that treatment is pursued immediately, and a WHO recommended dosage is taken within the required dosage time frame, then 1 / *a* is about 5 days (O’Meara et al. 2006). If the strain of malaria is not fully responsive to the drug, then *pa* measures the rate of clearing an infection via treatment where $0\le p <1$. If $p=0$, then the malaria strain is fully resistant to the drug and treatment is ineffective. For values of $0<p\le 1$, the resistant strain of malaria partially responds to treatment. We also assumed that asymptomatic and symptomatic infections of mature individuals are naturally cleared at the same rate ($\sigma _\mathrm{ma}=\sigma _\mathrm{ms}$), as in O’Meara et al. (2006), where a value of $1/33\ \hbox {days}^{-1}$ was used. Mean rates of immune-response-related clearance of $1/180\ \hbox {days}^{-1}$ have also been cited in Filipe et al. (2007). Here, we chose a baseline value based on a weighted average.

Our focus was on regions were malaria is holoendemic. These regions could either have low or high malaria transmission intensity. Low transmission intensity areas are typically upland sites (see, e.g., Craig et al. 1999) and tend to exhibit conditions that make them less conducive for the malaria transmitting mosquito to reproduce (Teboh-Ewungkem et al. 2014). Such conditions may include lower rainfall accumulations and cooler temperatures due to the altitude. Thus, with fewer mosquitoes, there are less contacts, on average, between humans and infectious female mosquitoes (O’Meara et al. 2006; Teboh-Ewungkem et al. 2014). On the other hand, high transmission regions, typically at lower elevations (Craig et al. 1999), have conditions that enhance the breeding and hence growth and reproduction of the female mosquito population. Thus, in high transmission regions, there is a higher on average contact between humans and infectious female mosquitoes (O’Meara et al. 2006; Teboh-Ewungkem et al. 2014). We used estimates from Chitnis et al. (2008) to inform our high and low mosquito biting, vector-to-host ratio, and transmission parameters.

Malaria mortality rates have been monitored since 2001 by Kenya Medical Research Institute (KEMRI) and the U.S. Centers for Disease Control and Prevention (CDC) as part of the KEMRI/CDC Health and Demographic Surveillance System (HDSS) in rural western Kenya (Desai et al. 2014). The results published in Desai et al. (2014) show a declining malaria disease-induced mortality rate in all age groups, with the 2010 data reported as 3.7 deaths per 1000 person-years for children under five, with a $95\%$ confidence interval reported to be between 3.0 and 4.5 per 1000 person-years. For individuals five and above, the malaria mortalities were estimated for 2010 as 0.4 deaths per 1000 person-years, with a $95\%$ confidence interval reported to be between 0.3 and 0.6 per 1000 person-years. The study appears to have accumulated the deaths yearly during the time frame used. The area of the study, around where KEMRI/CDC HDSS is located, is in the lake region of western Kenya, a malaria endemic region considered to be of high transmission intensity (Desai et al. 2014). For disease mortality in regions of low transmission intensity, we assume a 3.5 times reduction in the under five malaria-related mortality. This assumption comes from the findings in Snow and Omumbo (2006) that reported an approximately 3.5 times overall malaria-specific mortality in children in areas of higher stable transmission than in areas of low malaria transmission intensity in Sub-Saharan Africa, excluding southern Africa.

To initialize our simulations, we used a human density (in a $500\ \hbox {km}^2$ region of the KEMRI/CDC HDSS area the population density is 135,000 per $\hbox {km}^2$) and estimated mosquito density to be 3 times the human density for high transmission regions and 1 time the human density for low transmission regions (Amek et al. 2012). We assumed that both human and mosquito populations are constant in the absence of the disease, which implies equal birth and death rates for each species. Using the data in Table 4, we computed the human birth rate to be $\varLambda _\mathrm{h} = \frac{(\# \text {births per 1000 people per year})}{\text {1000 people}}\times \frac{1 \text { year}}{365 \text { days}}\times N_\mathrm{h}^*$ where $N_\mathrm{h}^*$ is the total human population. To keep the total population constant (apart from malaria deaths), the juvenile natural death rate was computed to be $\mu _\mathrm{h} = \frac{\varLambda _\mathrm{h}}{N_c^*} - \eta$ where $N_c^*$ is the total number of juveniles. Then, the mature death rate is $\mu _{mh} = \frac{\psi \eta }{1 - \psi }$ where $\psi =N_c^*/N_\mathrm{h}^*$ is the fraction of the population in the juvenile class.

The natural mosquito death rate, $\mu _v$, is assumed to be the reciprocal of the average lifetime of a mosquito. In the wild, mosquitoes are thought to live for about two weeks, though other modeling efforts have used values ranging up to 28 days (Ngonghala et al. 2012; Teboh-Ewungkem and Yuster 2010; Teboh-Ewungkem et al. 2010). We set the mosquito emergence rate to be $\varLambda _\mathrm{m} = \mu _v Q N_\mathrm{h}$, where *Q* is the number of mosquitoes per human. We assume the mosquito biting rate range to be $\alpha \in (0.2,0.5)$ per day (Mandal et al. 2011).

## Model Analysis

In this section, we derived the stability conditions of the disease-free equilibrium. We computed the basic reproduction number for the resistant and sensitive strains and present biological interpretations of the expressions. We also derived the invasion reproduction numbers and present invasion maps for the resistant and sensitive strains of malaria.

### The Disease-Free Equilibrium (DFE)

Let $\mathscr {X}=(I_\mathrm{s}, I_\mathrm{a}, J_\mathrm{s}, J_\mathrm{a}, I_\mathrm{ms}, I_\mathrm{ma}, J_\mathrm{ms}, J_\mathrm{ma}, M_\mathrm{s}, M_\mathrm{r}, S, T_\mathrm{s}, T, T_\mathrm{a}, R, S_\mathrm{m}, T_\mathrm{ms}, T_\mathrm{m}, T_\mathrm{ma}, R_\mathrm{m}, S_v)$ denote an equilibrium of the system described by (1a)–(1j), (2a)–(2j), and (4a)–(4c). The system has the DFE $\mathscr {E}_0=(0,0,0,0,0,0,0,0, 0,0, S_0, 0, T_0, 0, S_{m0}, 0, T_{m0}, 0, S_{v0} )$, where

6a $$\begin{aligned} S_0&=\frac{\varLambda _\mathrm{h}\left( r+\mu _\mathrm{h}+\eta \right) }{\left( \mu _\mathrm{h}+c+\eta \right) \left( r+\mu _\mathrm{h}+\eta \right) -rc}, ~~ T_0=\frac{c}{r+\mu _\mathrm{h}+\eta }S_0 \end{aligned}$$

6b $$\begin{aligned} S_{m0}&=\frac{\eta }{\mu _{mh}} \left( 1+\frac{rc}{\left( \mu _{mh}+r \right) \left( r+\mu _\mathrm{h}+\eta \right) } \right) S_0, \nonumber \\ T_{m0}&= \frac{\eta c}{\left( \mu _{mh}+r\right) \left( r+\mu _\mathrm{h}+\eta \right) }S_0, ~~~S_{v0} =\frac{\varLambda _v}{\mu _v}. \end{aligned}$$

**Table 4:** Data from Central Intelligence Agency (2013) on the three African countries, Kenya, Ghana, and Tanzania, used to determine current natural death rates and to infer death rates for malaria in our model

Data information	Kenya	Ghana	Tanzania
Total population	45,925,301	26,327,649	51,045,882
$< 5$ years old in millions	$\approx 3.3$	$\approx 1.9$	$\approx 4.1$
Infant mortality: deaths/1000 live births)	39.38	37.37	42.43
Births/1000 population	26.4	31.09	36.39
Deaths/1000 population	6.89	7.22	8
Life expectancy at birth in years	63.77	66.18	61.71
Calculated proportion under 5	0.0719	0.0722	0.0804

**Table 5:** Parameter values, ranges, and references that are unchanged across high/low transmission scenarios

Parameter	Value range	Baseline value	References
$\varLambda _\mathrm{h}$	$(2.24\times 10^3,5.08\times 10^3)$	$3.55 \times 10^3$	CIA data
$\mu _\mathrm{h}$	$(4.583\times 10^{-4},6.922\times 10^{-4})$	$5.94\times 10^{-4}$	CIA data
$\mu _\mathrm{mh}$	$(4.25\times 10^{-5},4.791\times 10^{-5})$	$4.43\times 10^{-5}$	CIA data
$\mu _{v}$	$\left( 1/21,1/7\right) \ \hbox {day}^{-1}$	$1/14\ \hbox {day}^{-1}$	Teboh-Ewungkem and Yuster (2010)
$\delta _\mathrm{m}$	$\left( \frac{0.3}{1000*365},\frac{0.6}{1000*365}\right) \ \hbox {day}^{-1}$	$\frac{0.4}{1000*365}\ \hbox {day}^{-1}$	Desai et al. (2014)
$\delta$	$\left( \frac{3.0}{1000*365}, \frac{4.5}{1000*365}\right) \ \hbox {day}^{-1}$	$\frac{3.7}{1000*365}\ \hbox {day}^{-1}$	Desai et al. (2014)
$1/\omega$	(28)	28 day	O’Meara et al. (2006)
$1/\omega '$	(370)	370 day	O’Meara et al. (2006)
$\nu$	$\left( 0.001, 0.05\right)$	0.01	O’Meara et al. (2006)
$\nu '$	$\left( 0.001, 0.05\right)$	0.05	O’Meara et al. (2006)
$\sigma _\mathrm{ms}$	(1/365–1/28)	$1/33\ \hbox {day}^{-1}$	Filipe et al. (2007), O’Meara et al. (2006)
$\sigma _\mathrm{ma}$	(1/365–1/28)	$0.03\ \hbox {day}^{-1}$	Filipe et al. (2007), O’Meara et al. (2006)
1 / *a*	(3, 10)	5 days	O’Meara et al. (2006)
*c*	(0.005, 0.03)	0.016 day$^{-1}$	O’Meara et al. (2006)
$1/r,1/r_\mathrm{s}$	Constant	1 / 6, $1/52\ \hbox {day}^{-1}$	O’Meara et al. (2006)

**Table 6:** Parameter values, ranges, and references that change across high/low transmission scenarios

Parameter	Value range	High baseline value	Low baseline value	References
$\varLambda _v$	$(1-10)*N_\mathrm{h}/\mu _v$	$3*N_\mathrm{h}/\mu _v$	$1*N_\mathrm{h}/\mu _v$	Chitnis et al. (2008), Amek et al. (2012)
$\beta _\mathrm{v}$	(0.03, 0.2)	0.0927	0.0313	Chitnis et al. (2008)
$\beta _\mathrm{h}$	(0.18, 0.9)	0.5561	0.1251	Chitnis et al. (2008)
$\kappa _{v}$	(0, 1)	0.6	0.6	Assumed
$\kappa _\mathrm{h}$	(0, 1)	0.6	0.6	Assumed
$\sigma _\mathrm{a}$	(1/365–1/20)	$1/33\ \hbox {day}^{-1}$	$1/180\ \hbox {day}^{-1}$	Filipe et al. (2007), O’Meara et al. (2006)
$\sigma _\mathrm{s}$	(0.02–0.05)	$0.03\ \hbox {day}^{-1}$	$1/365^{-1}$	Filipe et al. (2007), O’Meara et al. (2006)
*p*	(0, 1)	0.3	0.1	Assumed
$\lambda$	(0.25, 0.75)	0.5	0.7	O’Meara et al. (2006)
$\lambda '$	(0.15, 0.35)	0.2	0.7	O’Meara et al. (2006), Teboh-Ewungkem et al. (2014), Baliraine et al. (2009)
$\xi _\mathrm{m}$	(0.8, 1)	0.9	0.5	O’Meara et al. (2006), Teboh-Ewungkem et al. (2014), Baliraine et al. (2009)
$\xi$	(0.1, 0.5)	0.4	0.2	O’Meara et al. (2006), Teboh-Ewungkem et al. (2014), Baliraine et al. (2009)
*b*	(0.25, 0.50)	0.5	0.25	O’Meara et al. (2006)
$b_\mathrm{m}$	(0.25, 0.50)	0.5	0.25	O’Meara et al. (2006), Teboh-Ewungkem et al. (2014), Baliraine et al. (2009)
$\delta$		1.0137e−05	2.8963e−06	Desai et al. (2014)
$1/\eta$		5 years	8 years	Baliraine et al. (2009)

### Basic Reproduction Numbers 

The basic reproduction numbers for the sensitive parasite strain $\mathscr {R}_\mathrm{s}$ and the resistant parasite strain $\mathscr {R}_\mathrm{r}$ were computed using the next-generation matrix, as well as derived from biological interpretation of the model. Details of both approaches are listed in “Appendix B.” The reproduction number for the sensitive strain of infection takes the following form:

7 $$\begin{aligned} \mathscr {R}^2_\mathrm{s}= & {} \frac{\beta _v\beta _\mathrm{h}S_0S_{v0}}{\mu _vN_0^2} \left[ \frac{1-\lambda }{A_\mathrm{a}}+ \frac{\nu (1-\lambda )}{A_\mathrm{a}A_\mathrm{s}} + \frac{\eta \nu (1-\lambda )}{A_\mathrm{a}A_\mathrm{ms}A_\mathrm{s}} + \frac{\eta (1-\lambda )}{A_\mathrm{a} A_\mathrm{ma}}\right. \nonumber \\&\left. +\frac{\eta \nu '(1-\lambda )}{A_\mathrm{a}A_\mathrm{ma}A_\mathrm{ms}} +\frac{\lambda }{A_\mathrm{s}}+\frac{\eta \lambda }{A_\mathrm{s}A_\mathrm{ms}}\right] \nonumber \\&+\frac{\beta _v\beta _\mathrm{h}S_{m0}S_{v0}}{\mu _vN_0^2} \left[ \frac{1-\lambda '}{A_\mathrm{ma}}+ \frac{\nu '(1-\lambda ')}{A_\mathrm{ma}A_\mathrm{ms}} +\frac{\nu '}{A_\mathrm{ms}} \right] . \end{aligned}$$

The reproduction number for the resistant strain of infection takes the following form:

8 $$\begin{aligned} \mathscr {R}^2_\mathrm{r}= & {} \frac{\kappa _v\beta _v\kappa _\mathrm{h}\beta _\mathrm{h}(S_0+T_0)S_{v0}}{\mu _vN_0^2}\left[ \frac{1-\lambda }{B_\mathrm{a}}+ \frac{\nu (1-\lambda )}{B_\mathrm{a}B_\mathrm{s}} + \frac{\eta \nu (1-\lambda )}{B_\mathrm{a}B_\mathrm{ms}B_\mathrm{s}} + \frac{\eta (1-\lambda )}{B_\mathrm{a}A_\mathrm{ma}}\right. \nonumber \\&\left. +\frac{\eta \nu '(1-\lambda )}{B_\mathrm{a}A_\mathrm{ma}B_\mathrm{ms}} +\frac{\lambda }{B_\mathrm{s}} + \frac{\eta \lambda }{B_\mathrm{s}B_\mathrm{ms}}\right] \nonumber \\&+\frac{\kappa _v\beta _v\kappa _\mathrm{h}\beta _\mathrm{h}(S_{m0}+T_{m0})S_{v0}}{\mu _vN_0^2}\left[ \frac{1-\lambda '}{A_\mathrm{ma}} +\frac{\nu '(1-\lambda ')}{A_\mathrm{ma}B_\mathrm{ms}} +\frac{\nu '}{B_\mathrm{ms}} \right] . \end{aligned}$$

where the following parameters represent the durations of infections (see “Appendix B” for descriptions):

9 $$\begin{aligned} A_\mathrm{s}= & {} a+\mu _\mathrm{h}+\eta +\delta \qquad A_\mathrm{a} =c+\nu +\sigma _\mathrm{a}+\mu _\mathrm{h}+\eta \nonumber \\ A_\mathrm{ms}= & {} a+\mu _{mh}+\delta _\mathrm{m}+\sigma _\mathrm{ms} \qquad A_\mathrm{ma} =\nu '+\sigma _\mathrm{ma}+\mu _{mh} \nonumber \\ B_\mathrm{s}= & {} pa+\mu _\mathrm{h}+\eta +\delta \qquad B_\mathrm{a} =\nu +\sigma _\mathrm{a}+\mu _\mathrm{h}+\eta \nonumber \\ B_\mathrm{ms}= & {} pa+\mu _{mh}+\delta _\mathrm{m}+\sigma _\mathrm{ms} \end{aligned}$$

Note that for a mature individual, the duration of a resistant asymptomatic infection is equivalent to the duration of a resistant symptomatic infection ($1/A_\mathrm{ma}$).

**Fig. 4:**
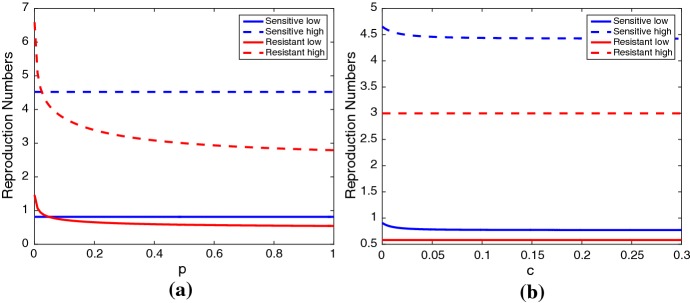
Reproduction numbers $R_\mathrm{s}$ (blue) and $R_\mathrm{r}$ (red) for the low transmission scenario (solid line) and high transmission scenario (dashed line) for varying values of **a***p* and **b***c*. All other parameter values are given in Tables 5 and 6 (Color figure online)

The reproduction numbers depend on the IPT treatment regime and drug efficacy (Fig. 4). The rate of IPT administration to individuals per day (*c*) has a small influence on $\mathscr {R}_\mathrm{s}$ (Fig. 4b). The drug efficacy (*p*) influences $\mathscr {R}_\mathrm{r}$ (Fig. 4a). For both low and high transmission scenarios, $\mathscr {R}_\mathrm{r}$ decreases for increasing levels of *p*. While increasing *p* decreases $\mathscr {R}_\mathrm{r}$, it is unable to bring $\mathscr {R}_\mathrm{r}<1$ in the high transmission scenario (Fig. 4a).

**Table 7:** Reproduction and invasion numbers for the low and high transmission scenarios using baseline parameter values from Tables 5 and 6

	Low transmission		High transmission			
	$\mathscr {R}_\mathrm{s}$	$\mathscr {R}_\mathrm{r}$	$\mathscr {R}_\mathrm{s}$	$\mathscr {R}_\mathrm{r}$	$\mathscr {R}^\mathrm{r}_\mathrm{s}$	$\mathscr {R}^\mathrm{s}_\mathrm{r}$
$r_\mathrm{s}=1/6$	0.8148	0.5811	4.5217	2.9984	1.329	4.533
$r_\mathrm{s}=1/52$	0.8148	0.5811	4.5217	2.9984	1.0821	6.7323

Since the low transmission basic reproduction numbers are less than one (so no sensitive- or resistant-only equilibria exist), we do not compute the invasion reproduction numbers

Table 7 presents the reproduction numbers for the sensitive strain, $\mathscr {R}_\mathrm{s}$, and resistant strain, $\mathscr {R}_\mathrm{r}$, using baseline parameter values for the low and high transmission scenarios in (7) and (8). In the low transmission scenario, both $\mathscr {R}_\mathrm{s}$ and $\mathscr {R}_\mathrm{r}$ are less than unity and malaria only persists in low transmission regions with regular introductions from outside. In the high transmission scenario, both $\mathscr {R}_\mathrm{s}$ and $\mathscr {R}_\mathrm{r}$ are greater than unity and malaria persists.

### Invasion Reproduction Numbers

The basic reproduction number is not sufficient to determine the competitive outcome of the resistant and sensitive strains. In addition to $R_\mathrm{s}$ and $R_\mathrm{r}$, we must derive the invasion reproduction numbers $R_\mathrm{r}^s$ and $R_\mathrm{s}^r$, which are threshold quantities determining whether the resistant strain is able to invade the sensitive-strain boundary equilibrium, and vice versa. The derivation follows the next-generation approach, but with the disease-free equilibrium replaced with either the sensitive-only boundary equilibrium, or the resistant-only boundary equilibrium.

The square of the thresholds determining whether the resistant strain can invade the sensitive-only boundary equilibrium, and whether the sensitive strain can invade the resistant-only boundary equilibrium, is given by:

10 $$\begin{aligned} (R_\mathrm{r}^s)^2= & {} \frac{\beta _v k_v S_v^*}{\mu _v N_\mathrm{h}^*}\cdot \frac{\beta _\mathrm{h} k_\mathrm{h}}{N_\mathrm{h}^*}\left\{ (S_\mathrm{m}^*+T_\mathrm{m}^*+T_\mathrm{ma}^*+T_\mathrm{ms}^*) \left[ \frac{(1 - \lambda ^\prime )}{A_\mathrm{ma}} + \frac{\lambda ^\prime }{B_\mathrm{ms}} + \frac{(1 - \lambda ^\prime )\nu ^\prime }{A_\mathrm{ma} B_\mathrm{ms}}\right] \right. \nonumber \\&+ (S^* + T_\mathrm{a}^* + T_\mathrm{s}^* + T^*)\left[ \frac{1 - \lambda }{B_\mathrm{a}} + \frac{\eta (1 - \lambda )}{A_\mathrm{ma} B_\mathrm{a}} + \frac{\lambda }{B_\mathrm{s}} + \frac{\eta \lambda }{B_\mathrm{ms} B_\mathrm{s}} + \frac{(1-\lambda ) \nu }{B_\mathrm{a} B_\mathrm{s}}\right. \nonumber \\&\left. \left. + \frac{\eta (1 - \lambda ) (A_\mathrm{ma}\nu + B_\mathrm{s} \nu ^\prime )}{A_\mathrm{ma} B_\mathrm{a} B_\mathrm{ms} B_\mathrm{s}}\right] \right\} \nonumber \\ (R_\mathrm{s}^r)^2= & {} \frac{\beta _\mathrm{h}\beta _v S_v^*}{\mu _v (N_\mathrm{h}^*)^2} \left\{ S_\mathrm{m}^*\left[ \frac{\lambda ^\prime }{A_\mathrm{ms}} + (1-\lambda ^\prime )\left( \frac{1}{A_\mathrm{ma}} + \frac{\nu ^\prime }{A_\mathrm{ms}A_\mathrm{ma}}\right) \right] \right. \nonumber \\&+ S^* \left[ \lambda \left( \frac{1}{A_\mathrm{ms}} + \frac{\eta }{A_\mathrm{s}A_\mathrm{ms}}\right) + (1-\lambda )\left( \frac{1}{A_\mathrm{a}} + \frac{\eta }{A_\mathrm{a}A_\mathrm{ma}} + \frac{\nu }{A_\mathrm{s}A_\mathrm{a}}\right. \right. \nonumber \\&\left. \left. \left. + \frac{\eta (A_\mathrm{ma}\nu + A_\mathrm{s}\nu ^\prime )}{A_\mathrm{s}A_\mathrm{a}A_\mathrm{ms}A_\mathrm{ma}} \right) \right] \right\} , \end{aligned}$$

where the equilibrium values correspond to the sensitive-only, and resistant-only boundary equilibria, respectively. Table 7 presents the invasion reproduction numbers ($\mathscr {R}^r_\mathrm{s}$, $\mathscr {R}^s_\mathrm{r}$) using baseline parameter values for the low and high transmission scenarios in (10). Here, the notation $X^*$ denotes the boundary equilibrium value of the state variable *X* (sensitive-only equilibrium for $R_\mathrm{r}^s$ and resistant-only equilibrium for $R_\mathrm{s}^r$).

## Numerical Results

In this section, we present results from numerical simulations for the high and low transmission regions. Our quantities of interest (QOI), or outputs, were number of children who died of malaria, number of adults who died of malaria, and the proportion of deaths that resulted from infection with the resistant strain. For both regions, we consider two IPT/treatment regimes: (1) SP/SP where SP, a long half-life drug (and could be replaced with another similar long half-life drug) is used for both IPT and treatment, and (2) SP/ACT where SP (the long half-life drug) is used for IPT and ACT, a short half-life drug (and could also be replaced by another similar short half-life drug such as AL), is used for treatment of symptomatic infection. We denote these scenarios as long/long and long/short. We also compute PRCC sensitivity indices for our outcomes to the parameters used. For simplification, and in an abundance of caution, we assume that the IPT drug and dose given are completely ineffective against the *resistant* pathogen when given to asymptomatic juveniles. The drug and dosages used for symptomatic treatment of the resistant pathogen, however, may be partially effective depending on the value chosen for *p*.

In this section, we demonstrate whether, and under which conditions, long half-life IPT should be used in combination with long or short half-life treatments and under which levels of effective treatment for resistant infections. Since IPT is currently being used in situations where the same long half-life drug is used both for prevention and treatment, we believe it is important to thoroughly show why this combination is detrimental under most situations. Since IPT efficacy is measured by child deaths prevented, and drug resistance is shown in terms of numbers of resistant strain infections compared to total infections, we present our results in terms of these two quantities.

For the following figures, we assume a high transmission region with an initial population of $N_\mathrm{h} = 35\cdot 10^6$ humans and a constant population of $105\cdot 10^6$ mosquitoes. Initial conditions: $N_{child}= 7.5\% N$, $S(0) = N_{child}$, $I_\mathrm{a}(0) = I_\mathrm{s}(0) = J_\mathrm{a}(0)=J_\mathrm{s}(0)=T(0) = T_\mathrm{a}(0) = T_\mathrm{s}(0) = R(0)=0$. For the adults, $N_{adult}=92.5\% N$, $S_\mathrm{m}(0)=53\% N_{adult}$, $I_\mathrm{ma}(0) = 10\% N_{adult}$, $I_\mathrm{ms}(0)=5\% N_{adult}$, $J_\mathrm{ma}(0) = J_\mathrm{ms}(0)=1\% N_{adult}$, $R_\mathrm{m}(0)=3 0\% N_{adult}$, with all other classes equal to zero. For the mosquitoes, we assume $S_v(0) = 90\% N_{mosquito}$, $M_\mathrm{r}(0)=M_\mathrm{s}(0)= 5\% N_{mosquito}$. We use these initial values to run the code without IPT for ten years (to a “pseudo-equilibrium”) to remove initialization effects in our numerical simulations. At this point, we then either continue the code with or without IPT.

**Fig. 5:**
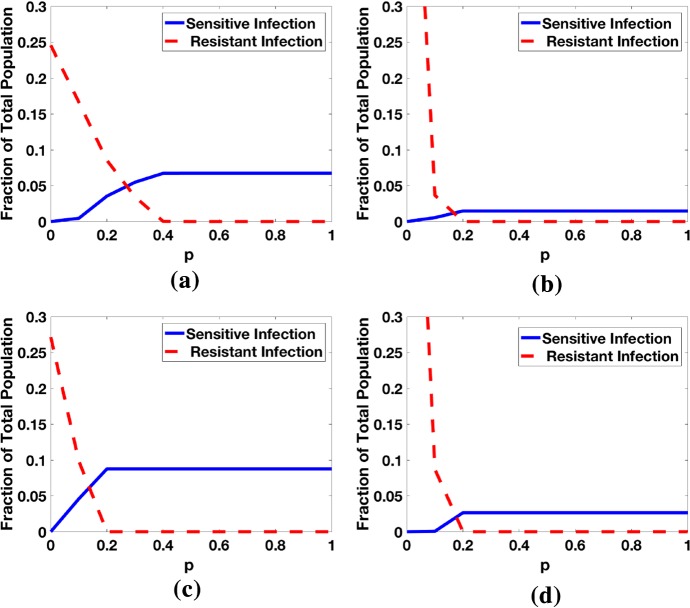
Fraction of the total population infected with sensitive and resistant strains at $t=10\ \hbox {years}$ when both treatment and IPT are applied the whole time. Note that the region for coexistence of the sensitive and resistant strains has a small range. As *p* increases, more people with the symptomatic resistant strain get effective treatment, thereby shortening the infectious period. The initial ratios for sensitive to resistant infections are different for the low transmission region because the initial prevalence of sensitive infections is low. **a** High transmission region (Long/long), **b** low transmission region (Long/long), **c** high transmission region (Long/short), **d** low transmission region (Long/short) (Colour figure online)

Figure 5 illustrates the effect of 10 years of IPT on the competition between the sensitive and resistant strain for different values of *p* using the long and short half-life treatments against symptomatic infection, and for high and low transmission regions. As the efficacy *p* of treatment against the resistant strain increases, the prevalence of the resistant strain decreases while the prevalence of the sensitive strain increases. If $p=0$, the resistant strain outcompetes the sensitive strain in both high and low transmission regions, regardless of the treatment drug half-life. In a high transmission region, the sensitive and resistant strains coexist for approximately $0<p<0.4$ when using the long half-life drug (Fig. 5a), and for approximately $0<p<0.2$ when using the short half-life drug (Fig. 5c). For the low transmission region (Fig. 5b, d), the resistant strain dominates until about $p=0.1$, at which point it drops precipitously while the sensitive strain increases for $0.1<p<0.2$ after which the resistant strain is extinct and the sensitive strain persists at low and steady levels due to treatment. The starting ratio is different for high and low transmission regions, which reflects the much higher prevalence of malaria, specifically the sensitive strain, in the high transmission regions.

### Numerical Results: High Transmission Region

#### Childhood Deaths Averted by IPT After 1, 5, and 10 Years

**Fig. 6:**
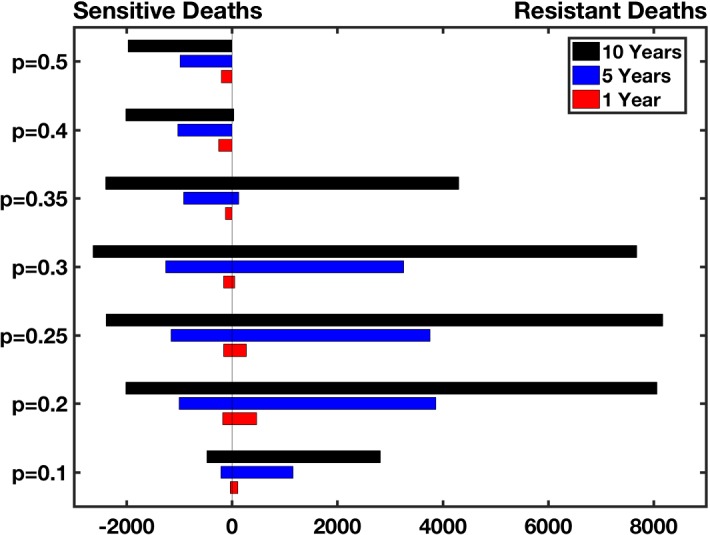
High transmission region: Net increase in deaths due to long half-life IPT usage, or (Total child deaths due to sensitive and resistant strains of malaria with IPT) − (total child deaths without IPT) for 1 year, 5 years, and 10 years of IPT use for different levels of standard treatment effectiveness against the resistant strain, *p*. The results for the long half-life treatment drug are broken into deaths averted due to sensitive infection and additional deaths due to resistant infection. IPT treatment can reduce the number of child deaths due to the sensitive infection, but increase the number of child deaths due to the resistant strain for some scenarios (Colour figure online)

As demonstrated in Fig. 6, IPT, along with a long half-life treatment drug for symptomatic infections (long/long scenario), decreases the number of childhood deaths due to the sensitive strain for $p\in \{0.1,0.2,0.25,0.3,0.4,0.5\}$, with the greatest reduction in deaths due to the sensitive strain occurring for $p=0.3$ after 10 years of IPT use. However, for $p\in {0.1,0.2,0.25}$, the reduction in deaths due to the sensitive strain after 1, 5, and 10 years of IPT use is dwarfed by the substantial increase in the number of deaths due to the resistant strain. When $p=0.3$, there is a benefit to using IPT for one year, with the reduction in sensitive deaths exceeding the increase in resistant deaths; however, at 5 and 10 years, the resistant strain has spread to the point where IPT is detrimental, increasing the total number of childhood deaths compared with the case when no IPT is used. For $p=0.4, 0.5$, the sensitive strain is dominant (as seen in Fig. 5) because high values of *p* reduce the duration of symptomatic, resistant infections (see expressions for $B_\mathrm{s}$ and $B_\mathrm{ms}$ in (9)), and therefore, IPT is able to be successful in averting total childhood deaths.

In the high transmission, long/short scenario, in which a short half-life drug is used for the treatment of symptomatic infections, IPT successfully reduced the number of childhood deaths for all values of *p*. After 1, 5, and 10 years of IPT, roughly 300–600, 2000–3000, and 4500–5100 childhood deaths were averted, respectively (see Table 9 in “Appendix C” for a summary of values for each *p*).

#### Interaction Between Efficacy of Resistant Treatment *p* and Time Between IPT Doses 1 / *c*

**Fig. 7:**
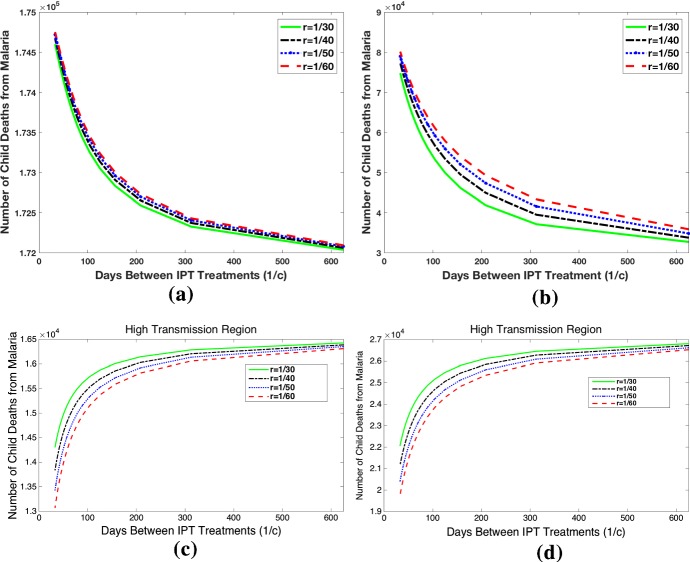
High transmission region: Total child deaths after 10 years of IPT for different intervals between IPT treatments, 1 / *c*, and for different values of 1 / *r*, the time chemoprophylaxis lasts in susceptible IPT-treated humans. (Top row: treatment effectiveness level $p=0.1$) For both the short and long half-life symptomatic treatment, any IPT will result in more resistance and more deaths for $p=0.1$. With the short half-life drug, the level of resistance and number of deaths is less than when long half-life is used for symptomatic treatment (long/long). (Bottom row: treatment effectiveness level $p=0.5$) In this case, both long and short half-life scenarios with IPT result in lives saved. However, since resistance is low, using the long half-life drug for symptomatic treatment is the best choice (saves more total lives). **a** Long/long scenario, $p= 0.1$, **b** Long/short scenario, $p=0.1$, **c** Long/long scenario, $p=0.5$, **d** Long/short scenario, $p=0.5$ (Colour figure online)

Figure 7 investigates how different rates of IPT treatments and treatment drug half-life influence the dynamics after 10 years. Figure 7a and b shows that in the high transmission region with $p=0.1$, the increase in time between IPT treatments, 1 / *c*, reduces the effects of malaria. In this scenario, the model predicts that the use of IPT has negative consequences, as the number of infections, childhood deaths, and proportion of resistant cases is high for low values of 1 / *c* regardless of the treatment drug half-life. Figure 7b and c shows that in the same scenario but with $p=0.5$, the use of IPT is beneficial. Here increasing time between IPT treatments, 1 / *c*, increases the number of infections and childhood deaths. In high transmission regions using long/long drug half-lives, we see that IPT should only be used for high values of *p*. We extend the time interval between IPT treatments to unrealistic lengths to show that there is no significant benefit to reducing drug resistance at the cost of extremely infrequent IPT treatments.

**Fig. 8:**
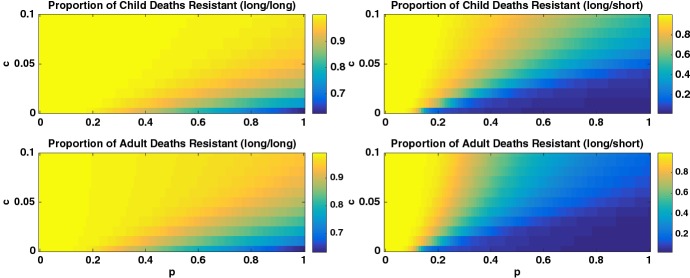
Heatmap of the *proportion* of deaths from the resistant strain for the high transmission region and for (left column: (long/long) scenario) long half-life drug for symptomatic treatment and (right column: (long/short) scenario) short half-life drug for symptomatic treatment. The top and bottom rows illustrate the proportion of child and adult deaths, respectively. Note different scales for the two columns. The proportion of deaths from the resistant strain is dependent on both *p* and *c*, showing that IPT schedule can increase resistance (Colour figure online)

The heatmaps in Fig. 8 illustrate the proportion of deaths in children and adults due to the resistant strain in a high transmission region as a function of *c* and *p* in both the long/long and long/short scenarios. We see that if both IPT and treatment have long half-lives (long/long), then the parameter space where the resistant strain dominates is much larger. When instead treatment has a short half-life (long/short), there is a wide range of parameter space for which the proportion resistant is low.

### Numerical Results: Low Transmission Region

For the low transmission region, we changed the parameters to match the low transmission parameters in Tables 5 and 6. For this scenario, the total number of child deaths from malaria is at least an order of magnitude smaller than in the high transmission region (see Fig. 6 and Table 9). In sheer numbers, then, IPT and treatment will have a lower impact in the low transmission region. The basic reproduction numbers for the sensitive and resistant strains are less than one at our low transmission baseline parameters (Table 7). Figure 4a shows that for very low values of *p*, indicating very high resistance to the treatment drug, the resistant strain has $R_\mathrm{r}>1$, larger than the sensitive strain reproduction number, $R_\mathrm{s}$. In Fig. 4b, the sensitive strain reproduction number is slightly reduced by *c* at very low values of *c*, corresponding to very infrequent IPT, but remains unchanged after that. The resistant reproduction number is unchanged by *c*. This means that frequency of IPT application has very little impact on either reproduction number for the low transmission region.

For $p>0.11$, IPT results in a net gain of lives saved for 1 year, 5 years, and 10 years for the long half-life drug used as treatment and as IPT. Past that point, in fact, there is very little difference across all values of *p*, unlike the high transmission scenario. However, as expected, the number of lives saved is an order of magnitude less than for the high transmission region. For $p<0.11$, application of IPT results in an increase in deaths over 5 and 10 years. There is a bifurcation point for *p* where the dominant strain switches from the sensitive to the resistant strain. Once the resistant strain is dominant, widespread use of the drug that it is resistant to leads to more, rather than fewer, deaths. When the short half-life drug is used for treatment and **long half-life drug** for IPT, we see a very similar bifurcation point at $p=0.11$ below which the resistant strain takes over and spreads, resulting in IPT being not only ineffective, but damaging. It is interesting to note that the increase in number of deaths from using IPT at $p=0.10$ for **short half-life** treatment is double the increase in deaths from IPT when **a long half-life drug** is used for treatment. This is in contrast to the high transmission region where using **a long half-life drug** as treatment results in a higher increase in deaths resulting from IPT usage. However, it should be noted that although the increase in deaths from using IPT is larger for **short half-life** treatment, the *total* number of deaths is larger when **a long half-life drug** is used for both treatment and IPT. See Fig. 6 and Table 9 for a summary of these results.

**Fig. 9:**
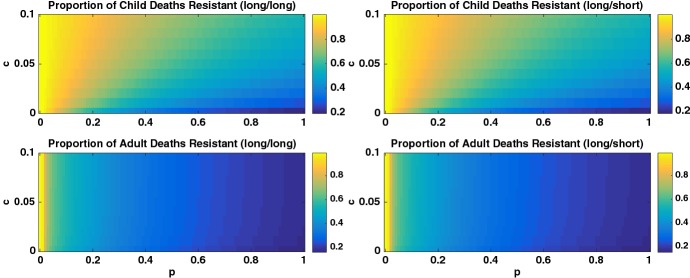
Heatmap of the *proportion* of deaths from the resistant strain for the low transmission region and for (left column: (long/long) scenario) long half-life drug for symptomatic treatment and (right column: (long/short) scenario) short half-life drug for symptomatic treatment. The top and bottom rows illustrate the proportion of child and adult deaths, respectively. Note different scales for the two columns. The proportion of deaths from the resistant strain is dependent on both *p* and *c*, showing that IPT schedule can increase resistance (Colour figure online)

Next we present heatmaps in Fig. 9 of the proportion of deaths from malaria across *p* and *c* space for the low transmission region for long/short and for long/long IPT/treatment half-lives. For both scenarios, the number of deaths depends almost exclusively on the value of *p* (efficacy of the treatment drug against resistant strain). However, the proportion of deaths from the resistant strain, as shown in Fig. 9, does depend on *c*, or the frequency of IPT doses, particularly as values of *p* increase. Also, unlike the high transmission region, the number of deaths from malaria in adults is unchanged by IPT usage.

We see in Fig. 6 and Table 9 that the total number of deaths of children from malaria increases dramatically as the value of *p* decreases for long/long drug half-lives. So, as strains develop more resistance to the drug used for treatment (low values of *p*), the number of deaths will increase if no new effective drug is available or put into use. For example, in the high transmission region, for $p=0.1$, there are nearly 10 times the number of deaths as for $p=0.5$. For high transmission regions, this effect is much more pronounced and occurs for higher values of *p*. For high transmission, number of deaths start drastically increasing for $p<0.3$, but for low transmission, this occurs for $p<0.11$. We can also see that IPT only results in significant ($>10\%$) reductions in total number of childhood deaths for $p>0.4$ and over 10 years in the high transmission region. For low transmission, if $p>0.11$, then a $>10\%$ reduction in child deaths occurs over 5 or more years. It is also interesting to note the distinctly nonlinear relationship between *p* and number of lives saved/lost due to IPT.

## Parameter Sensitivity

Latin hypercube sampling (LHS) (McKay et al. 1979), is a technique that uses stratified sampling without replacement. The LHS technique takes $n_p$ parameter distributions, divides them into *N* predetermined equally probable intervals, and then draws a sample from each interval. For the system described by (1a)–(1j), (2a)–(2j), and (4a)–(4c), with $n_p=18$ parameters, the technique generates a hypercube of size *N*, chosen to be 5000 row by 18 column matrix of parameter values. Each set of 18 parameter values is then used to generate a solution for the system given in (1a)–(1j), (2a)–(2j) and (4a)–(4c) for a total of 5000 simulations. The LHS method performs an unbiased estimate of the average model output, sampling each parameter interval shown as ranges in Tables 5 and 6 exactly once.

**Fig. 10:**
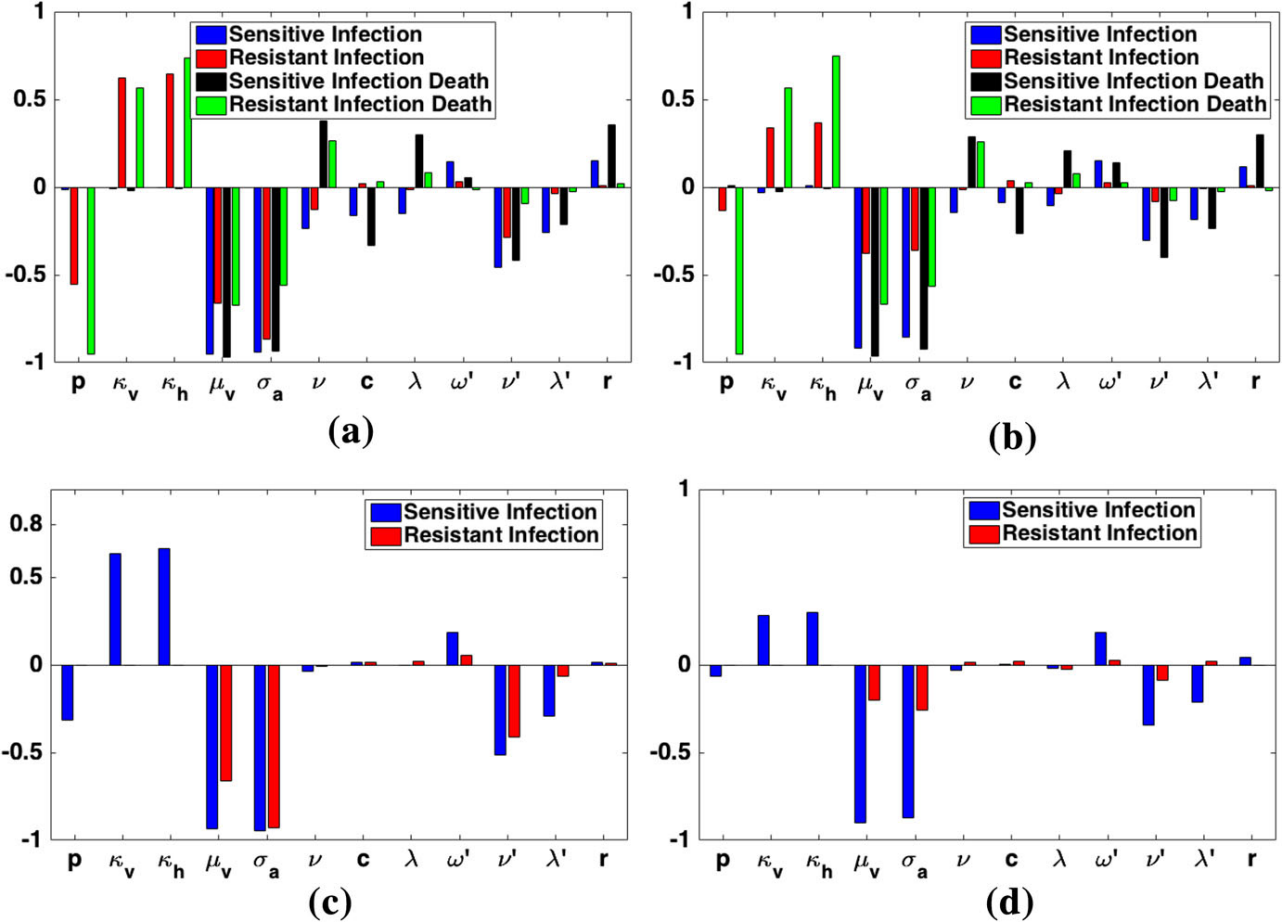
For **a**, **b** each parameter has a quartet of bars representing the PRCC values for sensitive child infections, resistant child infections, sensitive child deaths, and resistant child deaths. As time increases, the sensitivity to *p* decreases for resistant infections, but not for resistant deaths. However, there is little or no change to the sensitivity to $\mu _v$ and $\sigma _\mathrm{a}$ for the sensitive infections as well as the sensitive and resistant infections death between years 1 and 5; however, a marked decrease is seen for the resistant infections between years 1 and 5 indicating that a reduction in $\mu _v$ and $\sigma _\mathrm{a}$ would produce a corresponding decrease in the size of the number of resistant infections. For **c**, **d** each parameter has a doublet of bars representing the PRCC values for sensitive and resistant adult infections. As time increases, the sensitivity to *p*, $\kappa _v$ and $\kappa _\mathrm{h}$ decreases for sensitive infections. However, there is little or no change to the sensitivity to $\mu _v$ and $\sigma _\mathrm{a}$ for the sensitive infections but a marked decrease for the resistant infections. **a** Child, 1 year, **b** child, 5 years, **c** adult, 1 year, **d** adult, 5 years (Colour figure online)

Figure 10 shows only the statistically significant parameters (*p*-test value $< 0.01$). Note that as time increases from 1 to 5 years to 10 years since the start of IPT, the significance of *p* decreases for the sensitive and resistant infections. This is expected as the reproduction numbers $\mathscr {R}_\mathrm{S}$ and $\mathscr {R}_\mathrm{R}$ do not depend on *p*. However, the PRCC plot illustrates that the number of child deaths due to the resistant strain greatly decreases as *p* increases. This is a result we have seen repeatedly in our numerical simulations, illustrating that numerical simulations add to our understanding of the dynamical progression of IPT and its influence on death prevention and disease resistance. The PRCC plots for the high and low transmission regions show the same sensitivities as we have the same model for both regions with only changes in parameter values.

We can see in Fig. 10 that, for all QOI, $\mu _v$ and $\sigma _\mathrm{a}$, the death rate of mosquitoes and rate at which asymptomatic juveniles clear infection naturally, are extremely important. As the lifespan of the mosquito decreases (or $\mu _v$ increases), the QOI all decrease. As the time spent asymptomatic but still infectious for juveniles decreases (so $\sigma _\mathrm{a}$ increases), the QOI all decrease. There is little or no change to the sensitivity to $\mu _v$ and $\sigma _\mathrm{a}$ for the sensitive infections between years 1 and 5; however, a marked decrease is seen for the resistant infections between years 1 and 5 indicating that a reduction in $\mu _v$ and $\sigma _\mathrm{a}$ would produce a corresponding decrease in the size of the number of resistant infections. When we look at the sensitive and resistant infections death between years 1 and 5 for the child population, there is also little or no change to the sensitivity to $\mu _v$ and $\sigma _\mathrm{a}$. Thus, changes in $\mu _v$ and $\sigma _\mathrm{a}$ have similar impacts on the number of sensitive infections, as well as on the number of sensitive and resistant deaths for the child after the first year. By year 5, the number of resistant infections has dominated resulting in $\mu _v$ having a greater impact on resistant infections than sensitive infections. The same holds true for the parameter $\sigma _\mathrm{a}$. Consequently, although changes in $\mu _v$ and $\sigma _\mathrm{a}$ have a large impact on disease dynamics quantitatively, we do not expect our qualitative conclusions to change if we change the values of these parameters. Nonetheless, given their significance in Fig. 10, we further investigate their role on affecting the sensitive and infectious reproduction numbers and hence disease dynamics as a whole.

Without IPT, the role $\mu _v$ plays in malaria disease dynamics and control has been studied and reported, starting with Ross’s foundational work in 1911 (Ngonghala et al. 2015; Ross 1911; Teboh-Ewungkem et al. 2013). It has been shown that reducing the lifespan of the malaria transmitting mosquitoes will reduce disease incidence and malaria-related deaths. Thus, $\mu _v$, as a parameter for control, is fairly understood, and its upper bound value is about $\frac{1}{7}$ per day in the wild (in the laboratory, mosquitoes can be made to live longer). On the other hand, $\sigma _\mathrm{a}$, is not well understood in the absence of IPT but will impact the malaria disease dynamics. We now present a discussion of these parameters on the reproduction numbers, important epidemiological quantities for disease invasion and progression.

From the expressions and associated constants of the reproduction numbers in (7)–(9), the coordinates of the disease-free equilibrium in (6), and the expression for $\varLambda _{v}$ as stated on Table 6, it is clear that we can rewrite the reproduction numbers for the sensitive and resistant strains as $\mathscr {R}_\mathrm{s}^{2}=K_{1}/ \mu _{v}^{3}$ and $\mathscr {R}_\mathrm{r}^{2}=L_{1}/ \mu _{v} ^{3}$, respectively, where $K_{1}$ and $L_{1}$ are collections of variables independent of $\mu _{v}$. Thus, if we look at a local sensitivity of these reproduction numbers with respect to $\mu _v$, by computing the normalized sensitivity indices for each with respect to $\mu _{v}$, we get

11 $$\begin{aligned} \frac{\mu _{v}}{\mathscr {R}_\mathrm{s}^{2}}\frac{\partial \mathscr {R}_\mathrm{s}^{2}}{\partial \mu _{v}} =\frac{\left( \mu _{v}\right) ^{3}\cdot \mu _{v}}{K_1}\cdot \dfrac{-3K_1}{\left( \mu _{v}\right) ^{4}}=-3\ \text { and similarly} \frac{\mu _{v}}{\mathscr {R}_\mathrm{r}^{2}}\frac{\partial \mathscr {R}_\mathrm{r}^{2}}{\partial \mu _{v}}=-3. \end{aligned}$$

Thus, an increase in $\mu _{v}$ by say $10\%$ will yield similar percentage decrease ($30\%$) in the sensitive and resistant reproduction numbers. That is, the relative effect of $\mu _{v}$ on the sensitive and resistant reproduction numbers, important epidemiological parameters, will be similar.

As noted earlier, very little can be found in the literature on $\sigma _\mathrm{a}$, and thus, it is not well understood. However, it will impact the malaria disease dynamics as indicated by Fig. 10. In the presence of IPT, its impact on malaria diseases dynamics will be convoluted with the effects of IPT via the parameters *c* and *p*. In particular, from the same expressions and associated constants of the reproduction numbers in (7)–(9) and the coordinates of the disease-free equilibrium in (6), we can rewrite the reproduction numbers for the sensitive and resistant strains as functions of $\sigma _\mathrm{a}$ only. The expressions are $\mathfrak {R} _\mathrm{s}^{2}=S_{0}Z\left( \dfrac{K_{2}}{A_\mathrm{a}}+K_{3}\right) +S_{m0}ZK_{4}$ and $\mathfrak {R} _\mathrm{r}^{2}=(S_{0}+T_{0})\widetilde{Z} \left( \dfrac{L_{2}}{B_\mathrm{a}}+L_{3}\right) +(S_{m0}+T_{m0})\widetilde{Z}L_{4}$, respectively, where $\widetilde{Z}=\kappa _{\nu }\kappa _\mathrm{h}Z,$ and *Z*, $K_{i}$ and $L_{i}$ for $i=2,3,4$ are collections of variables independent of $\sigma _\mathrm{a}$, with $A_\mathrm{a}=c+\nu +\sigma _\mathrm{a}+\mu _\mathrm{h}+\eta$ and $B_\mathrm{a}=\nu +\sigma _\mathrm{a}+\mu _\mathrm{h}+\eta .$ Computing the sensitivity indices of these reproduction numbers with respect to $\sigma _\mathrm{a}$ yields

12 $$\begin{aligned} \frac{\sigma _\mathrm{a}}{ \mathfrak {R} _\mathrm{s}^{2}} \frac{\partial \mathfrak {R} _\mathrm{s}^{2}}{\partial \sigma _\mathrm{a}}= & {} \frac{S_{0} \left( -\dfrac{K_{2}}{A_\mathrm{a}^{2}}\right) \sigma _\mathrm{a}}{S_{0} \left( \dfrac{K_{2}}{A_\mathrm{a}}+K_{3}\right) +S_{m0}K_{4}}=\varsigma _{1}(c)\cdot \left( -\dfrac{\sigma _\mathrm{a}}{A_\mathrm{a}}\right) =-\varsigma _{1}(c) \left( \dfrac{\sigma _\mathrm{a}}{c+\sigma _\mathrm{a}+D}\right) , \nonumber \\ \end{aligned}$$

13 $$\begin{aligned} \frac{\sigma _\mathrm{a}}{ \mathfrak {R} _\mathrm{r}^{2}} \frac{\partial \mathfrak {R} _\mathrm{r}^{2}}{\partial \sigma _\mathrm{a}}= & {} \frac{(S_{0}+T_{0})\left( -\dfrac{L_{2}}{B_\mathrm{a}^{2}}\right) \sigma _\mathrm{a}}{(S_{0}+T_{0})\left( \dfrac{L_{2}}{B_\mathrm{a}}+L_{3}\right) +(S_{m0}+T_{m0})L_{4}}=\varsigma _{2}(c,p)\cdot \left( -\dfrac{\sigma _\mathrm{a}}{B_\mathrm{a}}\right) \nonumber \\= & {} -\varsigma _{2}(c,p)\left( \dfrac{\sigma _\mathrm{a}}{\sigma _\mathrm{a}+D}\right) , \end{aligned}$$

where $D=\nu +\mu _\mathrm{h}+\eta$, $\varsigma _{1}(c) = \frac{S_{0} \left( \dfrac{K_{2}}{A_\mathrm{a}}\right) }{S_{0}\left( \dfrac{K_{2}}{A_\mathrm{a}}+K_{3}\right) +S_{m0}K_{4}}$ and $\varsigma _{2}(c,p) = \frac{(S_{0}+T_{0})\left( \dfrac{L_{2}}{B_\mathrm{a}}\right) }{(S_{0}+T_{0})\left( \dfrac{L_{2}}{B_\mathrm{a}}+L_{3}\right) +(S_{m0}+T_{m0})L_{4}}$. Both $\varsigma _{1}(c)$ and $\varsigma _{2}(c,p)$ lie in the interval [0, 1] and are dependent on other parameters notably the IPT-related parameters *c* and *p*. Thus, in the presence of IPT, the IPT-related parameters would convolute the impact of $\sigma _\mathrm{a}$ on the sensitive and resistant parasite strains and hence disease control, when all other parameters are held constant. However, if $c=0$, i.e., no IPT, and $p=1$, then $\varsigma _{1}(0)=\varsigma _{2}(0,1)$ and the effects are again similar on both the sensitive and resistant strain.

Thus, based on the calculations of the normalized sensitivity indices (a local sensitivity metric) computed along with the initial discussion about what these normalized indices and global sensitivity results imply under changes in $\mu _v$ and $\sigma _\mathrm{a}$, we argue that our overall message from a qualitative standpoint will remain unchanged under changes in these two parameters $\mu _v$ and $\sigma _\mathrm{a}$ when IPT is administered.

Additional important parameters are *p*, $\kappa _v$, and $\kappa _\mathrm{h}$. The number of child deaths from resistant infection is particularly sensitive to *p* and as *p* increases, that number decreases. $\kappa _v$, and $\kappa _\mathrm{h}$ are measures of the competitive disadvantage of the resistant strain. As they increase toward 1 (so the competitive disadvantage decreases), the resistant infections and resistant deaths increase significantly.

## Discussion and Conclusion

There are a few general patterns in our simulations. First, using a short half-life treatment drug, assumed here to be effective against both sensitive and resistant symptomatic infections, decreases the advantage of the resistant strain, so also reduces the dependence of resistant emergence on IPT. Second, all the results are highly sensitive to *p*, and the value of *p* at which the resistant strain dominates depends on whether it is a low or high transmission region. There are strong nonlinear relationships between *p*, *c*, and the IPT and treatment drug half-lives. There are bifurcations in realistic parameter regimes that suggest IPT should be applied with caution and with a good knowledge of the background levels of resistance in the region. Finally, we specifically considered both short- and long-range results (1–10 years) to inform the sustainability of current IPT and treatment programs. Particularly as new drugs are not developed quickly, it will be important to know if our current protocols will result in high levels of resistance in the future.

In the high transmission region, successful invasion of resistant strains is mostly driven by the drug(s) used for symptomatic treatment. Over the first year, IPT has a 0.1–5% effect (both increases and reductions) on the total number of deaths from malaria for all scenarios. When a short half-life drug such as AL or ACT is used for treatment, IPT usage always results in lives saved with a 16.5–18.5% reduction in total child deaths over 5 years (around 4500–5000 lives saved). However, when a long half-life drug such as SP is used for symptomatic treatment, use of IPT results range from a 13% increase in deaths to an 8.5% decrease in deaths over 5 years (from 2900 additional deaths to 1000 lives saved). When resistance to the treatment drug is high (*p* is low), then IPT use results in faster takeover of the resistant strain, thus causing in more deaths. The few studies available considering the role of IPT in resistance provide mixed results, which is in line with our model output. In Mali, after one year, the use of IPTi did not show an increase in molecular markers of resistance (Dicko et al. 2010). However, in a region in Tanzania with widespread SP resistance, use of IPTp was shown to significantly increase levels of resistance (Harrington et al. 2009). Initially, then, one would then recommend using a short half-life treatment drug whenever possible while applying IPT with a long half-life drug such as SP and closely monitoring levels of resistance.

However, it is important to note the effect that the half-life of the symptomatic treatment drug has on total number of deaths. In particular, a short half-life treatment drug gives very similar total number of deaths across the resistance level spectrum, from partially to nearly fully resistant. The long half-life drug used as treatment gives order of magnitude differences in total deaths depending on the level of resistance. When $p=0.10$ (resistance is high), there are 119,000 total deaths over 5 years, whereas when $p = 0.50$ (low resistance) there are about 11,000 deaths over 5 years. For the short half-life treatment drug scenario, the total number of deaths over 5 years is about 17,000 for all levels of resistance considered and thus gives much lower number of deaths than the long/long scenario for highly resistant strains, but higher total deaths if resistance is weak.

The take-home message is that (1) treatment drugs are generally driving resistance in high transmission areas and the role of IPT in driving resistance tends to be minor comparatively, (2) however, when a highly resistant strain is circulating, IPT can indeed result in increased levels of resistance and loss of lives, particularly over longer time periods, and (3) in general, when short half-life drugs such as AL or ACT are used for treatment and SP is used for IPT, as is currently the case, regular use of IPT in children will result in potentially thousands of lives saved over the course of 5–10 years. We point out that the dynamics can be complex, so there are levels of resistance for which IPT saves lives over a short time period, but results in a cumulative loss of lives over 5–10 year periods as resistance levels ramp up. Therefore, our model suggests caution in using IPT without a corresponding heightened surveillance and awareness of changes in the circulating resistant strains over time. If resistance were to be significantly increasing over time, then evaluation of both the treatment drug and IPT usage would be warranted. Finally, we measured the effectiveness of IPT in lives saved. There may also be other benefits, such as a shortened length of asymptomatic malaria infections, that are not measured here.

In low transmission regions, we see different patterns in the costs and benefits of IPT. Here, IPT can have a much larger role in driving resistance when highly resistant strains are circulating. For example, in the long/long scenario with a highly resistant strain circulating, the proportion of resistant cases stays low when IPT is not used, but rises to over 70% in children over the course of 10 years when IPT is used (Fig. 8). For the long/short scenario, IPT also results in an increase in proportion resistant that would not otherwise occur, but at a greatly reduced rate of increase (Fig. 8). However, for all but the most highly resistant strains, IPT usage in low transmission regions results in lives saved and does not drive take over of resistant strains. IPT generally results in a 24–26% reduction in deaths in the long/long scenario over 5 years (about 120 lives saved) and in 26–29% decrease in deaths for the long/short scenario over 5 years (about 140 lives saved). Thus, in general, it is better to use the short half-life treatment drug with a long half-life IPT in the low transmission regions. Although it is not as critical as in the high transmission regions, our model does suggest some caution and an increased awareness of circulating resistant strains is warranted when IPT is used in a low transmission region.

A more complete cost–benefit analysis that includes cost of IPT and treatment drugs per dose, total number of doses needed, and a broader definition of benefits including not only deaths averted but severe and asymptomatic cases averted and reductions in total time infected would be interesting. We have not considered how IPT might directly change the age at which children gain the “mature” status based on a combination of many previous exposures to malaria and general improvement in the immune system due to age. Effective use of IPT could in fact increase that age, resulting in more serious cases of malaria in older than usual children. This could result again in increases of deaths or serious disease in what we are now calling the mature age group. We have focused solely on the use of SP as the IPT drug while varying the drugs used for treatment. While this is generally true currently, considering additional drugs for potential use as IPT could be useful. We are looking at holoendemic regions with no seasonality (year-round transmission), and it would be interesting to extend to regions with seasonal malaria transmission. Studying the interaction between vector control measures, which would impact $\mu _v$ and $\sigma _\mathrm{a}$, IPT, and treatment, is an interesting subject for future work as well.

## Appendix A

See Table 8.

**Table 8:** Duration (in months) of asymptomatic parasitemia by age and microgeographic locale; prevalence of asymptomatic malaria by age and region; and percent of vector population found in each locale

	Age	Valley bottom	Middle hill	Hilltop	Asymp. prevalence
Altitude in meters (Village)		1430 (Iguhu)	1500 (Makhokho)	1580 (Sigalagala)	
Duration (in months) of parasitemia by age	Age 5–9	6	4	3	34.4%
	Age 10–14	6	4	3	34.1%
	Age $> {14}$	1	1	1	9.1%
% asymptomatic by region		52.4%		23.3%	
% of vectors found in region		98%	1%	1%	
% of 334 asymptomatic episodes in region		44%	24.9%	31.1%	

This region is considered hypoendemic. 15% of asymptomatic episodes lasted 1 month. 38.1% of episodes lasted 2–5 months, and 14.2% of episodes lasted 6–12 months. 32.5% experienced no infection episode. Iguhu is near the Yala River, a major breeding site for *An. gambiae* mosquitoes (Baliraine et al. 2009)

## B Basic Reproduction Numbers

The basic reproduction numbers for the sensitive parasite strain $\mathscr {R}_\mathrm{s}$ and the resistant parasite strain $\mathscr {R}_\mathrm{r}$ were computed using the next-generation matrix. The next-generation matrix (NGM) is

$$\begin{aligned} K= & {} \begin{pmatrix} 0 &{} K_{1,2}\\ K_{2,1} &{} 0 \end{pmatrix}, \qquad \text{ where } \\ K_{1,2}= & {} \begin{pmatrix} \frac{\beta _\mathrm{h} \lambda S0}{\mu _\mathrm{m} N_0} &{} 0 &{} 0 &{} 0 &{} 0 &{} 0 \\ \frac{\beta _\mathrm{h} (1-\lambda ) S_0}{\mu _\mathrm{m} N_0} &{} 0 &{} 0 &{} 0 &{} 0 &{} 0 \\ 0 &{} \frac{\beta _\mathrm{h} k_\mathrm{h} \lambda (S_0+T_0)}{\mu _\mathrm{m} N_0} &{} 0 &{} 0 &{} 0 &{} 0 \\ 0 &{} \frac{\beta _\mathrm{h} k_\mathrm{h} (1-\lambda ) (S_0+T_0)}{\mu _\mathrm{m} N_0} &{} 0 &{} 0 &{} 0 &{} 0 \\ \frac{\beta _\mathrm{h} \lambda ' S_{m0}}{\mu _\mathrm{m} N_0} &{} 0 &{} 0 &{} 0 &{} 0 &{} 0 \\ \frac{\beta _\mathrm{h} (1-\lambda ') S_{m0}}{\mu _\mathrm{m} N_0} &{} 0 &{} 0 &{} 0 &{} 0 &{} 0 \\ 0 &{} \frac{\beta _\mathrm{h} k_\mathrm{h} \lambda _p (S_{m0}+T_{m0})}{\mu _\mathrm{m} N_0} &{} 0 &{} 0 &{} 0 &{} 0 \\ 0 &{} \frac{\beta _\mathrm{h} k_\mathrm{h} (1-\lambda ') (S_{m0}+T_{m0})}{\mu _\mathrm{m} N_0} &{} 0 &{} 0 &{} 0 &{} 0 \end{pmatrix} \quad \text{ and } \\ K_{2,1}= & {} \begin{pmatrix} k_{9,1} &{}k_{9,2} &{} 0 &{} 0 &{} k_{9,5} &{} k_{9,6} &{} 0 &{} 0 \\ 0 &{} 0 &{} k_{10,3}&{} k_{10,4}&{} 0 &{} 0 &{} k_{10,7} &{}k_{10,8}\\ 0 &{} 0 &{} 0 &{} 0 &{} 0 &{} 0 &{} 0 &{} 0\\ 0 &{} 0 &{} 0 &{} 0 &{} 0 &{} 0 &{} 0 &{} 0\\ 0 &{} 0 &{} 0 &{} 0 &{} 0 &{} 0 &{} 0 &{} 0\\ 0 &{} 0 &{} 0 &{} 0 &{} 0 &{} 0 &{} 0 &{} 0 \end{pmatrix}. \\ k_{9,1}= & {} \frac{\beta _\mathrm{m} S_{v0}}{N_0}\left( 1+\frac{\eta }{A_\mathrm{ms}}\right) \quad k_{9,2}= \frac{\beta _\mathrm{m} S_{v0}}{A_\mathrm{a} N_0}\left( 1+\frac{\nu }{A_\mathrm{s}} + \frac{\eta (A_\mathrm{ma} \nu +A_\mathrm{s} \nu ')}{A_\mathrm{s} A_\mathrm{ms} A_\mathrm{ma}} +\frac{ \eta }{A_\mathrm{ma}}\right) \\ k_{9,5}= & {} \frac{\beta _\mathrm{m} S_{v0}}{A_\mathrm{ms} N_0} \quad k_{9,6}= \frac{\beta _\mathrm{m} S_{v0}}{A_\mathrm{ma} N_0} \left( 1+\frac{\nu '}{A_\mathrm{ms}}\right) \\ k_{10,3}= & {} \frac{\beta _\mathrm{m} k_\mathrm{m} S_{v0}}{B_\mathrm{s} N_0} \left( 1+\frac{\eta }{B_\mathrm{ms}}\right) \\ k_{10,4}= & {} \frac{\beta _\mathrm{m} k_\mathrm{m} S_{v0}}{B_\mathrm{a} N_0} \left( 1+\frac{ \eta (A_\mathrm{ma} \nu +B_\mathrm{s} \nu ')}{B_\mathrm{s} A_\mathrm{ma} B_\mathrm{ms}} + \frac{\eta k_\mathrm{m}}{A_\mathrm{ma}} + \frac{\nu }{B_\mathrm{s}} \right) \\ k_{10,7}= & {} \frac{\beta _\mathrm{m} k_\mathrm{m} S_{v0}}{B_\mathrm{ms} N_0} \quad k_{10,8} = \frac{b_\mathrm{m} k_\mathrm{m} S_{v0}}{A_\mathrm{ma} N_0} \left( 1+\frac{\nu '}{B_\mathrm{ms}}\right) \end{aligned}$$

In addition to the next-generation matrix approach, the reproduction numbers were derived based on the biological interpretation of the model.

### *Sensitive Reproduction Number*$\mathscr {R}_\mathrm{s}$

Let $\mathscr {R}_\mathrm{s-asym}^\mathrm{naive}$ and $\mathscr {R}_\mathrm{s-sym}^\mathrm{naive}$ denote the reproduction numbers for the sensitive strain of infection associated with asymptomatic and symptomatic cases in naive humans, respectively. Let $\mathscr {R}_\mathrm{s-asym}^\mathrm{mature}$ and $\mathscr {R}_\mathrm{s-sym}^\mathrm{mature}$ denote the reproduction numbers for the sensitive strain of infection associated with asymptomatic and symptomatic cases, in mature humans, respectively.

At the beginning of an outbreak, the proportion of the population susceptible to the sensitive parasite is $S_0+S_{m0}$. A portion of this sensitive population will become asymptomatically infected and either remain asymptomatic or transition to a symptomatic case (there is no transition from symptomatic to asymptomatic in this model). A portion of these infected individuals will age into the mature population. The sensitive reproduction number for the asymptomatic cases in the naive population over the full course of infection, i.e., the number of naive human asymptomatic cases resulting from one initially sensitive case, is then given by

The sensitive symptomatic reproduction number for the naive population, or the number of naive human cases resulting from one initial symptomatic individual, is given by

The sensitive reproduction number for the asymptomatic cases in the mature population over the full course of infection, i.e., the number of mature human asymptomatic cases resulting from one initially sensitive case, is then given by

The sensitive symptomatic reproduction number for the mature population, or the number of mature human cases resulting from one initial symptomatic individual, is given by

Then, the reproduction number for the sensitive strain of infection takes the following form:

14 $$\begin{aligned} \mathscr {R}^2_\mathrm{s}= & {} \mathscr {R}_\mathrm{s-asym}^\mathrm{naive} +\mathscr {R}_\mathrm{s-sym}^\mathrm{naive}+ \mathscr {R}_\mathrm{s-asym}^\mathrm{mature} +\mathscr {R}_\mathrm{s-sym}^\mathrm{mature} \nonumber \\= & {} \frac{\beta _\mathrm{m}\beta _\mathrm{h}S_0S_{v0}}{\mu _\mathrm{m}N_0^2} \left[ \frac{1-\lambda }{A_\mathrm{a}}+ \frac{\nu (1-\lambda )}{A_\mathrm{a}A_\mathrm{s}} + \frac{\eta \nu (1-\lambda )}{A_\mathrm{a}A_\mathrm{ms}A_\mathrm{s}} + \frac{\eta (1-\lambda )}{A_\mathrm{a}A_\mathrm{ma}}\right. \nonumber \\&\left. +\frac{\eta \nu '(1-\lambda )}{A_\mathrm{a}A_\mathrm{ma}A_\mathrm{ms}} +\frac{\lambda }{A_\mathrm{s}} +\frac{\eta \lambda }{A_\mathrm{s}A_\mathrm{ms}} \right] \nonumber \\&+\frac{\beta _\mathrm{m}\beta _\mathrm{h}S_{m0}S_{v0}}{\mu _\mathrm{m}N_0^2} \left[ \frac{1-\lambda '}{A_\mathrm{ma}}+ \frac{\nu '(1-\lambda ')}{A_\mathrm{ma}A_\mathrm{ms}} +\frac{\nu '}{A_\mathrm{ms}} \right] . \end{aligned}$$

The above reproduction number $\mathscr {R}_\mathrm{s}$ was also computed using the next-generation matrix approach.

### *Resistant Reproduction Number*$\mathscr {R}_\mathrm{r}$

Let $\mathscr {R}_\mathrm{r-asym}^\mathrm{naive}$ and $\mathscr {R}_\mathrm{r-sym}^\mathrm{naive}$ denote the reproduction numbers for the resistant strain of infection associated with asymptomatic and symptomatic cases in naive humans, respectively. Let $\mathscr {R}_\mathrm{r-asym}^\mathrm{mature}$ and $\mathscr {R}_\mathrm{r-sym}^\mathrm{mature}$ denote the reproduction numbers for the resistant strain of infection associated with asymptomatic and symptomatic cases, in mature humans, respectively.

At the beginning of an outbreak, the proportion of the population susceptible to the resistant parasite is $S_0+S_{m0}+T_0+T_{m0}$. The resistant reproduction number for the asymptomatic cases in the naive population over the full course of infection, i.e., the number of naive human asymptomatic cases resulting from one initially resistant case, is then given by

The resistant symptomatic reproduction number for the naive population, or the number of naive human cases resulting from one initial symptomatic individual, is given by

The resistant reproduction number for the asymptomatic cases in the mature population over the full course of infection, i.e., the number of mature human asymptomatic cases resulting from one initially resistant case, is then given by

The resistant symptomatic reproduction number for the mature population, or the number of mature human cases resulting from one initial symptomatic individual, is given by

Then, the reproduction number for the resistant strain of infection takes the following form:

15 $$\begin{aligned} \mathscr {R}^2_\mathrm{r}= & {} \mathscr {R}_\mathrm{r-asym}^\mathrm{naive} +\mathscr {R}_\mathrm{r-sym}^\mathrm{naive}+ \mathscr {R}_\mathrm{r-asym}^\mathrm{mature} +\mathscr {R}_\mathrm{r-sym}^\mathrm{mature} \nonumber \\= & {} \frac{\kappa _\mathrm{m}\beta _\mathrm{m}\kappa _\mathrm{h}\beta _\mathrm{h}(S_0+T_0)S_{v0}}{\mu _\mathrm{m}N_0^2} \left[ \frac{1-\lambda }{B_\mathrm{a}}+ \frac{\nu (1-\lambda )}{B_\mathrm{a}B_\mathrm{s}} + \frac{\eta \nu (1-\lambda )}{B_\mathrm{a}B_\mathrm{ms}B_\mathrm{s}} + \frac{\eta (1-\lambda )}{B_\mathrm{a}A_\mathrm{ma}}\right. \nonumber \\&\left. +\frac{\eta \nu '(1-\lambda )}{B_\mathrm{a}A_\mathrm{ma}B_\mathrm{ms}} +\frac{\lambda }{B_\mathrm{s}}+\frac{\eta \lambda }{B_\mathrm{s}B_\mathrm{ms}}\right] \nonumber \\&+\frac{\kappa _\mathrm{m}\beta _\mathrm{m}\kappa _\mathrm{h}\beta _\mathrm{h}(S_{m0}+T_{m0})S_{v0}}{\mu _\mathrm{m}N_0^2} \left[ \frac{1-\lambda '}{A_\mathrm{ma}}+ \frac{\nu '(1-\lambda ')}{A_\mathrm{ma}B_\mathrm{ms}} +\frac{\nu '}{B_\mathrm{ms}} \right] . \end{aligned}$$

The above reproduction number $\mathscr {R}_\mathrm{r}$ was also computed using the next-generation matrix approach.

## C Total Child Deaths

In Table 9, we see that the resistant strain only dominates after introduction in the low transmission region for very low values of *p*, which equates to very high resistance to the drug used for treatment in the resistant strain. For the **long/long** IPT/treatment half-life scenario, the total number of deaths jumps by more than a factor of 3 when $p=0.09$. For the **long/short** scenario, a smaller jump in cases is seen at $p=0.09$. In absolute numbers, IPT saves more lives in the high transmission region, but as a percent reduction of total deaths, IPT does better in the low transmission region. Another interesting pattern is that for higher values of *p*, using short half-life treatment results in more deaths than using long half-life treatment. However, once a highly resistant strain is circulating, the long/short regime has lower total deaths than long/long. For example, in the low transmission region, when $p=0.10$, there are 1599 deaths without IPT and 1836 deaths with IPT for long/long after 5 years. By contrast, for long/short there were 698 deaths without IPT and 1060 deaths with IPT. If a very resistant strain is circulating, it is better to use a short half-life treatment drug.

**Table 9:** Total number of child deaths from malaria for various values of *p* and either no IPT or IPT used

*p*	Year 1		Year 5		Year 10	
	No IPT	IPT	No IPT	IPT	No IPT	IPT
*High transmission region, long/long*						
0.1	77,743	77,823	118,505	119,455	171,716	174,047
0.2	26,806	27,103	43,929	46,795	67,258	73,304
0.25	14,860	14,973	25,622	28,221	40,901	46,687
0.3	9158	9052	14,772	16,771	24,720	29,759
0.35	8533	8407	12,167	11,375	16,959	18,864
0.4	8394	8141	12,021	10,990	16,717	14,742
0.5	8254	8052	11,878	10,888	16,575	14,605
*Low transmission region, long/long*						
0.09	2309	2308	4950	4961	9179	9192
0.1	696	684	1599	1836	4125	4930
0.11	323	303	503	379	787	497
0.12	313	295	495	373	784	503
0.13	300	281	482	359	772	490
0.15	301	285	484	363	774	494
0.2	288	270	471	348	760	479
0.3	279	268	462	346	751	477
*High transmission region, long/short*						
0.1	13,500	13,147	19,596	17,080	27,522	22,776
0.2	13,341	12,955	19,440	16,714	27,367	22,292
0.25	13,275	12,842	19,371	17,134	27,318	22,822
0.3	13,235	12,572	19,331	16,847	27,258	22,425
0.35	13,208	12,649	19,304	16,762	27,230	22,508
0.4	13,187	12,856	19,284	16,784	27,212	22,424
0.5	13,164	12,793	19,260	17,068	27,187	22,678
*Low transmission region, long/short*						
0.09	1539	1536	3741	3866	7688	7877
0.10	499	765	698	1060	1137	2658
0.11	380	366	650	459	940	622
0.12	358	340	572	428	915	577
0.13	349	326	563	415	906	563
0.15	340	323	554	411	896	559
0.2	329	316	543	404	886	552
0.3	321	300	535	388	878	537

The (IPT/treatment) half-lives are also noted where the long half-life drug is SP and the short half-life drug is (AL). For high resistance to treatment (low values of *p*), the total number of deaths is much higher than for lower resistance to treatment. The cutoff for this dramatic increase in number of deaths is at about $p=0.3$ for the high transmission region and about $p=0.1$ for the low transmission region for long/long scenario

## Footnotes

1 This assumption comes from evidence in Cravo et al. (2001) suggesting higher success in parasite clearance under some background immunity. We note, however, that the original study was performed on the rodent malaria *Plasmodium chabaudi*, where it was shown that drug-resistant parasites could be cleared in partially immune individuals.
